# Compound 4f, a novel brain-penetrant reversible monoacylglycerol inhibitor, ameliorates neuroinflammation, neuronal cell loss, and cognitive impairment in mice with kainic acid-induced neurodegeneration

**DOI:** 10.1371/journal.pone.0312090

**Published:** 2024-11-21

**Authors:** Naoto Arimura, Chie Maeda, Kazunobu Aoyama, Namiko Yamaguchi, Ayumu Sugiura, Yasuko Takahashi, Ryouta Maeda, Tatsuya Ando, Makoto Kamata, Hideki Matsui, Maiko Tanaka

**Affiliations:** 1 Neuroscience Drug Discovery Unit, Research, Takeda Pharmaceutical Company Limited, Kanagawa, Japan; 2 DMPK, Research, Takeda Pharmaceutical Company Limited, Fujisawa, Kanagawa, Japan; 3 Computational Biology, Research, Takeda Pharmaceutical Company Limited, Fujisawa, Kanagawa, Japan; University of Modena and Reggio Emilia, ITALY

## Abstract

Neuroinflammation, a hallmark of neurodegenerative diseases, is associated with neuronal cell loss and cognitive dysfunction. Monoacylglycerol lipase (MAGL) is involved in neuroinflammation in the brain via the degradation of endocannabinoid 2-arachidonoylglycerol to arachidonic acid, a precursor of some eicosanoids; therefore, MAGL inhibitors are expected to have anti-inflammatory effects. We recently developed a reversible, selective, central nervous system penetrant, and orally available MAGL inhibitor, compound **4f**. Compound **4f** (1 mg/kg) robustly increased 2-arachidonoylglycerol levels and decreased arachidonic acid levels in the mouse brain. To examine whether compound **4f** can suppress neuroinflammation and neuronal cell loss, kainic acid (KA)-injected mice were used as a neuroinflammation model in this study. Compound **4f** (1 mg/kg) significantly decreased the cytokine and chemokine expression levels and suppressed neuronal cell loss in the hippocampi of mice. Compound **4f** also ameliorated cognitive impairment in KA-injected mice. The cannabinoid receptor 1 antagonist, AM251, and cannabinoid receptor 2 antagonist, AM630, partly blocked the neuroprotective effects of compound **4f** in the hippocampi of KA-injected mice. Gene expression profiles and pathway analyses revealed that compound **4f** reversed the KA-induced changes in the expression of genes related to inflammation and neurotransmission. These results indicate that the selective and reversible MAGL inhibitor, compound **4f**, can be used as a potential therapeutic agent for the treatment of neurodegenerative diseases.

## Introduction

Neuroinflammation is one of the most significant hallmarks of neurodegenerative diseases, including Alzheimer’s disease (AD) and Parkinson’s disease (PD) [1]. Activated microglia increase the expression levels of inflammatory markers, including interleukin (IL)-1β and tumor necrosis factor-alpha (TNF-α), in AD [2], and chronic activation of microglia is implicated in neurotoxicity and synapse loss by triggering several proinflammatory cascades. These results indicate that neuroinflammation is involved in the progression of neurodegenerative diseases.

Endocannabinoid signaling is a key pathway in neuroinflammation. The endocannabinoid signaling system is composed of cannabinoid receptors 1 (CB1R) and 2 (CB2R) as well as their endogenous ligands, such as 2-arachidonoylglycerol (2-AG) and anandamide (AEA). CB1R and CB2R are mainly expressed in the neurons and microglia, respectively. Although the currently available findings regarding changes in CB1R expression are inconsistent [3, 4], increased CB2R expression levels in the postmortem brains of patients with AD has been reported [5]. Treatment with a CB1R agonist exerts neuroprotective effects in a rodent model of neurodegeneration [6], indicating that CB1R activation has beneficial effects on neuroinflammation and neuronal loss in neurodegenerative conditions. Similarly, CB2R activation attenuates spatial memory impairment and neurodegeneration in rodents [7]. Moreover, 2-AG has anti-inflammatory and neuroprotective effects [8–12], and 2-AG levels are increased in the plasma of patients with AD. This may be a compensatory mechanism that protects against neurodegenerative decline [13]. In contrast, arachidonic acid (AA), a metabolite of 2-AG and AEA, is a precursor for eicosanoid synthesis and activation of the AA cascade exacerbates the AD pathology [14]. AA metabolism may be increased in AD brains [15], although changes in AA levels largely depend on food intake rather than pathogenesis. These findings indicate that the endocannabinoid system activated by 2-AG and AEA via CB1R or CB2R contributes to anti-inflammatory and neuroprotective effects.

Monoacylglycerol lipase (MAGL) is a member of the serine hydrolase family, which hydrolyzes monoacylglycerol to fatty acids and glycerol [16]. MAGL is a major hydrolase of 2-AG in the brain [17]. Pharmacological or genetic inactivation of MAGL reduces 2-AG hydrolysis activity and increases brain 2-AG levels in rodents [18]. Therefore, MAGL inhibitors may ameliorate neuroinflammation and neuronal cell death via CB1 and CB2 receptor activation by increasing the 2-AG levels. An irreversible MAGL inhibitor, JZL184, significantly protects against dopaminergic neurodegeneration and dopamine loss in the 1-methyl-4-phenyl-1,2,3,6-tetrahydropyridine model of PD [19]. When considering MAGL inhibitors as drug candidates, reversible inhibition of MAGL can reduce the risk of consecutive and chronic activation of the endocannabinoid system, which is related to the brain reward system, appetite, and memory. However, whether reversible MAGL inhibition with a small molecule can exert anti-inflammatory and neuroprotective effects remains unclear.

We previously discovered a novel reversible MAGL inhibitor, compound **4f**, which showed high potency and good selectivity over closely related serine hydrolases, including fatty acid amide hydrolase (FAAH) and alpha/beta hydrolase-6 (ABHD6), along with low human ether-a-go-go-related gene liability [20].

This study aimed to determine the *in vivo* pharmacological efficacy of a reversible MAGL inhibitor, compound **4f**, in a mouse model of neuroinflammation and neurodegeneration. As a mouse model of neurodegeneration, kainic acid (KA), an analog of glutamate, was used to induce neuronal damage accompanied by inflammatory responses in the brain [21, 22]. Here, we demonstrated that compound **4f** repressed KA-induced neuroinflammation and neuronal cell loss in the hippocampi of mice, while improving the cognitive impairment in the same animal model. To elucidate its mechanism of action, we investigated the gene expression profiles in treated mice and the effects of cannabinoid receptor antagonists on neuroprotection. Changes in the expression of genes related to inflammation and neurotransmission in KA-treated mice were inhibited by compound **4f**. Moreover, the CB1R antagonist, AM251, and CB2R antagonist, AM630, partly blocked the protective effects of compound **4f** in the hippocampi of KA-treated mice. These results suggest that the reversible MAGL inhibitor compound **4f** has therapeutic potential for the treatment of neurodegenerative diseases.

## Materials and methods

### Animals

Male C57BL/6J mice (6–7-weeks-old) were obtained from Charles River Laboratories (Yokohama, Japan). The animals were housed in a light-controlled room (12-h light/dark cycle, the light turns on at 7AM) food and water provided *ad libitum*) and habituated for at least one week before starting the experiments. All animal research protocols used in this study were approved by the Institutional Animal Care and Use Committee of Takeda Pharmaceutical Company Limited.

### Drugs

The drugs, 2s,4s)-2-((3-((3-chloro-4-methylbenzyl)oxy)zetidine-1-yl)carbonyl)-7-oxa-5-azaspiro[3.4]octan-6-one (compound **4f** in [20]) and T-211 (compound 8 in [23], were synthesized by Takeda Pharmaceutical Company Limited. Compound **4f** was suspended in 0.5% (w/v) methylcellulose in distilled water and were administered to mice orally at a volume of 10 mL/kg in all experiments. T-211 was dissolved in dimethylacetamide in a saline solution. KA monohydrate was purchased from Sigma-Aldrich and was dissolved in saline. AM251, a CB1R antagonist, and AM630, a CB2R antagonist, were purchased from Cayman Chemical and dissolved in vehicle containing 5% ethanol, 5% Cremophor EL, and 90% saline.

### Measurement of compound 4f pharmacokinetics

Seven-week-old mice were orally administered compound **4f** (1 mg/kg). Plasma and brain samples were collected at 0.5, 1, 2, 4, 8, and 24 h post-treatment (n = 4 per group). The cerebral hemispheres were homogenized in 4-fold saline (v/w). The samples of plasma and brain homogenates were deproteinized by addition of acetonitrile containing diclofenac as the internal standard. After centrifugation, the supernatants were subjected to LC-MS/MS analysis, using an UFLC system (Shimadzu Co. Ltd, Kyoto, Japan) coupled to an API 5000 triple quadrupole mass spectrometer (SCIEX, MA) and a Shim-pack XR-ODS, C18 column (20 mm × 2.0 mm, 5 μm, Shimadzu Co. Ltd). The flow rate and column temperature were set at 0.7 mL/min and 50°C, respectively. The mobile phases A and B consisted of 10 mmol/L ammonium formate supplemented with formic acid (100:0.2, v/v) and acetonitrile with formic acid (100:0.2, v/v), respectively. The total run time was set at 1.6 min. The initial concentration of mobile phase B was set at 5%. The condition was maintained for 0.1 min, followed by a linear increase in the mobile phase B up to 95% in 0.1 min. The condition was held for 0.8 min. The condition was returned to its initial state and held for 0.6 min for re-equilibration.

### Assessment of compound 4f target occupancy

Nine-week-old mice were orally administered compound **4f** at 0.1, 0.3, 1, 3, and 10 mg/kg 0.5 h prior to intravenous administration of tracer T-211(0.03 mg/kg). These mice were decapitated 0.5 h after T-211 administration to obtain the plasma and brain samples. Cortices and hippocampi were quickly dissected and frozen on dry ice. The plasma and brain concentrations of compound **4f** and T-211 were measured in the LC-MS/MS method above. The apparent target occupancy (TO) of compound **4f** was calculated based on the Kp values of the tracer, according to the following equation: apparent TO (%) = (Kp of T-211 in vehicle-treated group − Kp of T-211 in compound **4f** treated group of x mg/kg)/(Kp of T-211 in vehicle-treated group − Kp/T-211 in compound **4f** treated group of 10 mg/kg) × 100.

### Measurement of 2-AG and AA levels in the brains of mice

Detailed procedure of 2-AG and AA measurement has been described in a previous report [24]. Briefly, brain samples from mice treated with compound **4f** were collected and frozen rapidly on dry ice. Cerebral hemispheres were homogenized in 9-fold (v/w) isopropanol and centrifuged. The supernatant was mixed with internal standard solutions (AA-d8 and 2-AG-d8 in isopropanol). The sample solution was then subjected to LC-MS/MS analysis. The samples of plasma and brain homogenates were deproteinized by addition of acetonitrile containing diclofenac as the internal standard. After centrifugation, the supernatants were subjected to LC-MS/MS analysis, using an UFLC system (Shimadzu Co. Ltd, Kyoto, Japan) coupled to an API 5000 triple quadrupole mass spectrometer (SCIEX, MA) and a Shim-pack XR-ODS, C18 column (20 mm × 2.0 mm, 5 μm, Shimadzu Co. Ltd). The flow rate and column temperature were set at 0.7 mL/min and 50°C, respectively. The mobile phases A and B consisted of 10 mmol/L ammonium formate supplemented with formic acid (100:0.2, v/v) and acetonitrile with formic acid (100:0.2, v/v), respectively. The total run time was set at 1.6 min. The initial concentration of mobile phase B was set at 5%. The condition was maintained for 0.1 min, followed by a linear increase in the mobile phase B up to 95% in 0.1 min. The condition was held for 0.8 min. The condition was returned to its initial state and held for 0.6 min for re-equilibration.

### KA administration and drug treatment

The outline of the experimental design was described in S1 File. On the first day of the experiment, compound **4f** (0.1, 0.3, and 1 mg/kg) was administered to seven-week-old mice. One hour after compound **4f** administration, the mice were anesthetized with pentobarbital sodium (50 mg/kg, intraperitoneal; Somnopentyl®, Kyoritsu Seiyaku, Tokyo, Japan) and fixed to a stereotaxic apparatus (Kopf Instruments, Tujunga, CA, USA). For intracerebroventricular (i.c.v.) administration of KA (0.2 μg), an injection cannula was implanted into the left lateral ventricle (0.2 mm posterior to bregma, 1.0 mm lateral to the midline, 2.0 mm depth from the skull surface). After i.c.v. injection with KA, the mice were kept warm on hot plate until recovering from anesthesia, and were confirmed to exert no behavioral abnormalities, then returned to normal breeding environment. When mice showed score 5 (continuous rearing and falling) or score 6 (severe tonic-clonic seizure with loss of postural control) in a modified Racine scale [25] after KA treatment, euthanasia were performed for those mice using carbon dioxide gas immediately. Compound **4f** was administered to the mice once daily for three days. On day 4, the mice were subjected to the behavioral test or dissected by decapitation 24 h after the last administration of compound **4f**, and the hippocampi were quickly collected. In the experiments involving co-administration of CB1R (AM251) and CB2R (AM630), the antagonist was administered intraperitoneally 1 h prior to compound **4f** treatment.

### RNA extraction, cDNA synthesis, and reverse transcription-quantitative real-time polymerase chain reaction (RT-qPCR)

Total RNA was isolated using Qiazol lysis reagent (QIAGEN) and RNeasy columns (QIAGEN) and the total RNA was reverse transcribed into cDNA before performing qPCR. qPCR was performed using the TaqMan Master Mix (Applied Biosystems, MA, USA) and primer-probe sets on a QuantStudio7 instrument (Applied Biosystems). Relative quantification of gene expression was performed using glyceraldehyde 3-phosphate dehydrogenase (GAPDH) as the endogenous control gene using the 2^−ΔΔCT^ method. Primer-probe sets were purchased from Applied Biosystems. TNF-α: Mm00443258_m1, IL-1β: Mm00434228_m1, IL-6: Mm01210732_g1, Gapdh: Mm99999915_g1.

### RNA sequencing and bioinformatics analysis

RNA quality was assessed using an Agilent 2100 Bioanalyzer system with an RNA 6000 Nano Assay kit (Agilent Technologies, CA, USA). Libraries were constructed using Dynabeads mRNA DIRECT Micro KIT, Magnetic Bead Cleanup Module, Ion RNA-Seq Core Kit/Primer Set v2, and Ion Xpress RNA-Seq Barcode 01–16 Kit (Life Technologies, CA, USA). The final library quality and quantity were analyzed using an Agilent high-sensitivity DNA assay (Agilent Technologies). Equimolar pooling of libraries was performed using an Ion Proton^TM^ System (Life Technologies).

The sequence reads were mapped to the mouse genome (mm10), and read counts were obtained. Genes with average counts of less than 2.7 across all samples were excluded from the analysis. Differentially expressed genes (DEGs) were identified using voom [26] after trimming the mean of M-value normalization [27]. *P*-values were adjusted for multiple comparisons using the Benjamini–Hochberg false discovery rate. Genes were considered differentially expressed if |fold change| ≥ 1.2 and adjusted *P*-value < 0.05. The DEG lists were uploaded to Ingenuity Pathway Analysis (IPA) (QIAGEN), and the biological pathways enriched in DEGs were analyzed.

### Measurement of monocyte chemotactic protein-1 levels in the hippocampi of mice

The hippocampi were homogenized in RIPA buffer (Pierce) containing cOmplete^TM^, Mini, EDTA-free protease inhibitor cocktail (Roche), and PhosSTOP^TM^ phosphatase inhibitor cocktail (Roche). The lysates were centrifuged at 10,000 rpm at 4°C for 10 min. The concentration of monocyte chemotactic protein-1 (MCP-1) was measured using the MCP-1 mouse/rat ELISA Kit Quantikine (R&D Systems).

### Tissue processing and immunohistochemistry

Mice were deeply anesthetized with pentobarbital sodium (50 mg/kg, intraperitoneal, Somnopentyl®, Kyoritsu Seiyaku) and transcardially perfused with heparinized saline, followed by phosphate-buffered 4% paraformaldehyde (pH 7.4) under anesthesia. The brains were extracted and post-fixed overnight in 4% paraformaldehyde. The tissue samples were embedded in optimal cutting temperature compound after 30% sucrose/PBS replacement. Coronal sections around the bregma (between 1.5 and 2.3 mm posterior to the bregma) were prepared, and these sections were incubated in 10 mM citrate buffer (pH 6.0) at 98°C for 20 min for epitope retrieval. Subsequently, they were treated with 0.3% hydrogen peroxide solution to block endogenous peroxidase, followed by a blocking reagent (Protein Block, Serum Free, Blocking, Unconjugated, Liquid form, Immunohistochemistry, code: x0909, DAKO, Eugene, OR, USA) before staining. The sections were probed with primary antibodies against NeuN (1:50000 dilution; rabbit monoclonal, code: ab177487, Abcam), followed by incubation with horseradish peroxidase-conjugated polymer, EnVision+ (code: K4003, DAKO), and 3,3′-diaminobenzidine. The stained sections were mounted after hematoxylin counterstaining. Terminal deoxynucleotidyl transferase dUTP nick end labeling (TUNEL) assays were performed with Apop Tag Peroxidase In situ Apoptosis Detection Kit (Merck/Millipore, MA, USA). All the slide results were digitized using a Nanozoomer 2.0-HT slide scanner (Hamamatsu Photonics, Japan). Digital Pathology Quantification analyses of NeuN-positive areas and TUNEL-positive neuronal cells in the right hippocampus were performed using WinROOF2015 (MITANI, Japan). Quantitative analysis of the NeuN-positive areas was performed in the CA1 and CA3 regions of the hippocampus. The TUNEL-positive neuronal cells in the hippocampus were counted.

### Novel object recognition test (NORT)

A gray box of 30 × 30 × 25 cm was used as the test box, and a silver-colored aluminum cylinder and a white-colored ceramic triangular pyramid were used as objects. In the acquisition session on day 4 after the KA treatment, two identical objects were placed symmetrically in the two corners of the test box. A mouse was placed in another corner of the box with its head toward the corner. The time spent exploring each object was recorded for 5 min, followed by a 24-h retention interval in the home cage. On day 5, the same mice were placed again individually into the same box, except that one of the familiar objects used during the acquisition test was replaced with a novel object. Mice were allowed to explore freely for 5 min. The novelty discrimination index (NDI) was calculated using the following equation: novel object interaction/total interaction × 100 (%). Mice that exhibited no interaction with each object in the acquisition session were not tested in the retention session and mice that showed no interaction with each object in the retention session were excluded from the data. The results were obtained from two independent experiments conducted over two consecutive weeks.

### Statistical analyses

Statistical analyses were performed using the Exsus software (CAC Croit Corporation, Japan). Student’s *t*-test or the Aspin-Welch test was performed to assess the statistically significant differences between the two groups. Differences were defined as statistically significant at *p* ≤ 0.05. In experiments with multiple doses of compound **4f**, statistical significance was analyzed using one-tailed Williams’ test. In experiments with the combination of compound **4f** and CB1R and CB2R antagonists, statistical significance was analyzed using the Steel’s or Dunnett’s multiple comparison test after confirming the statistically significant differences between the saline- and KA-injected groups. Data are presented as the mean ± standard deviation or mean ± standard error of the mean. Differences were considered to be statistically significant at *p* ≤ 0.05.

## Results

### Pharmacokinetics and pharmacodynamics of compound 4f in mice

**Fig 1:**
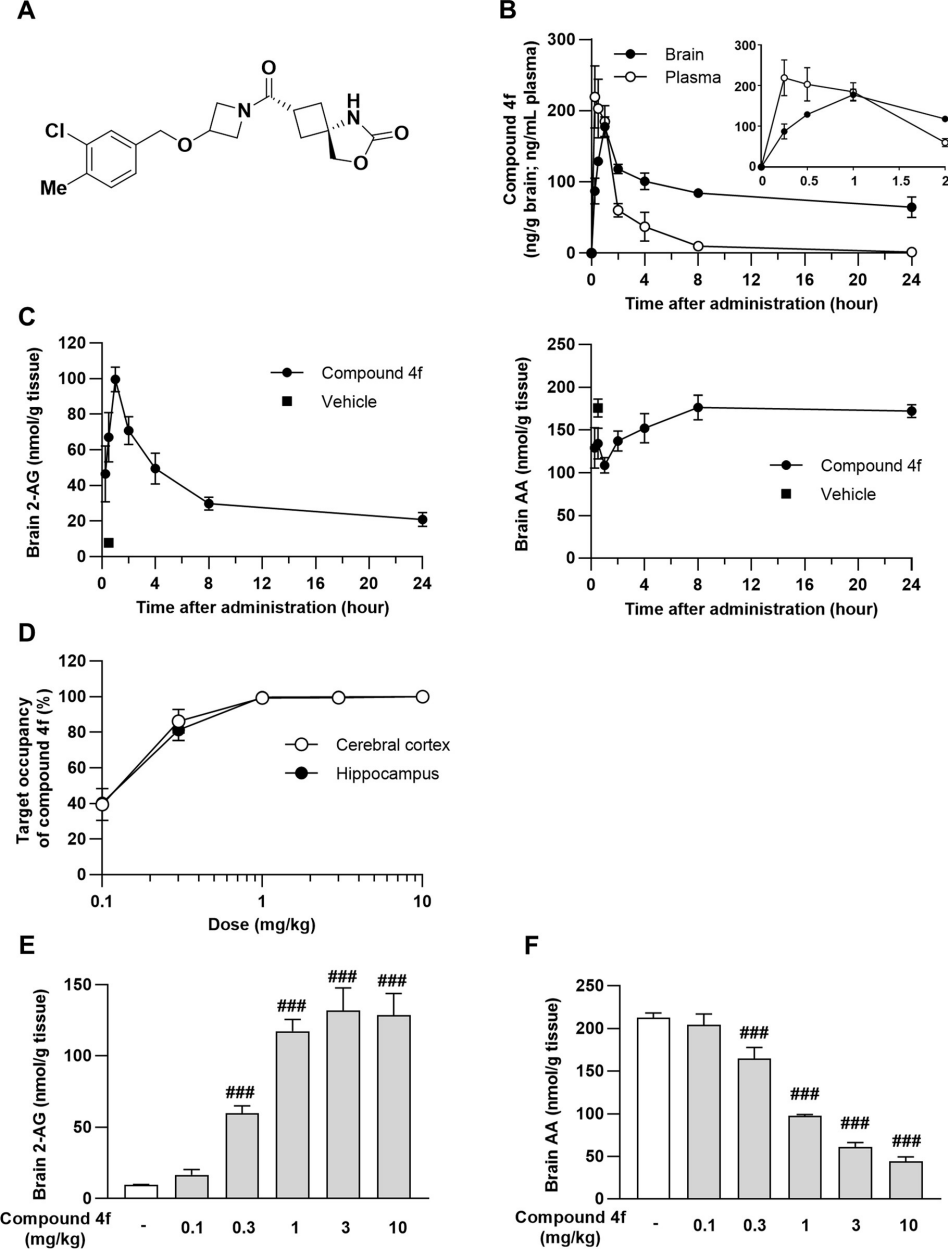
Pharmacokinetics and pharmacodynamics of compound 4f in mice. (A) Chemical structure of compound **4f**. (B) Concentrations of compound **4f** in the plasma and brain samples following oral administration of 1 mg/kg. Mean ± standard deviation (SD), n = 4. The inset graph indicates the compound **4f** concentration up to 2 h. (C) Brain 2-arachidonoylglycerol (2-AG) and arachidonic acid (AA) levels determined at the indicated time points in brain hemispheres. Compound **4f** was administered orally at 1 mg/kg. Mean ± SD, n = 4. (D) Occupancy rate of compound **4f** in the hippocampi and cerebral cortices of mice 1 h after oral treatment. Compound **4f** at 0.03, 0.1, 0.3, 1, and 10 mg/kg was administered orally followed by the administration of T-211 after 0.5 h. The target occupancy of compound **4f** at 10 mg/kg was determined as 100%. Mean ± SD, n = 3. (E–F) Brain 2-AG (E) and AA (F) levels. Compound **4f** at 0.03, 0.1, 0.3, 1, and 10 mg/kg was administered orally to mice. Mean ± SD, n = 3. *###P* ≤ 0.001 compared to vehicle-treated mice (Williams’ test). 2-AG, 2-arachidonoylglycerol; AA, arachidonic acid; KA, kainic acid; SD, standard deviation.

A novel reversible MAGL inhibitor, compound **4f** (Fig 1A), has been described previously [20]. Compound **4f** was a potent MAGL inhibitor (human half-maximal inhibitory concentration [IC_50_] = 6.2 nM, mouse IC_50_ = 13 nM) with high selectivity over FAAH and ABHD6 (IC_50_ > 10,000 nM). In the present study, the selectivity profile of compound **4f** (10 μM) was confirmed on a panel of 109 radioligand binding and functional assays comprising enzymes, ion channels, and receptors, which revealed that compound **4f** displayed less than 50% inhibition in all but three of the tested assays: monoamine oxidase-B (50%), serotonin (5-hydroxytryptamine) 2B (81%), and non-selective sigma type receptor (62%) (Eurofins Cerep Panlabs Taiwan, Ltd.; S1 Table). The previous study [20] demonstrated that compound 4f increased brain 2-AG dose-dependently and the 2-AG level reached up to approximately maximal level by treatment with compound 4f at 1 mg/kg. Here, pharmacokinetics of compound **4f** was examined until 24 h after single dose at 1 mg/kg. The brain concentration of compound **4f** (1 mg/kg) reached its maximum 1 h after oral treatment in mice (Fig 1B). Compound **4f** robustly increased brain 2-AG levels by approximately 14-fold 1 h after administration compared to that in the vehicle-treated group (Fig 1C). Concomitantly, compound **4f** decreased the brain AA levels (Fig 1C). Notably, the time course of changes in brain 2-AG levels reflected the brain pharmacokinetics of compound **4f** (Fig 1B and 1C).

To understand the occupancy of MAGL by compound **4f** at 1 mg/kg, we conducted a competition assay using compounds **4f** and T-211, which belongs to a chemical series distinct from compound **4f** and was described as compound 8 in a previous study [23]. The occupancy ratio of compound **4f** increased in a dose-dependent manner and reached a maximum of over 99% at 1 mg/kg (Fig 1D). The result of occupancy reflected a dose-dependent increase in brain 2-AG and a decrease in brain AA levels (Fig 1E and 1F).

### Effects of compound 4f on neuroinflammation in mice with KA-induced neurodegeneration

**Fig 2:**
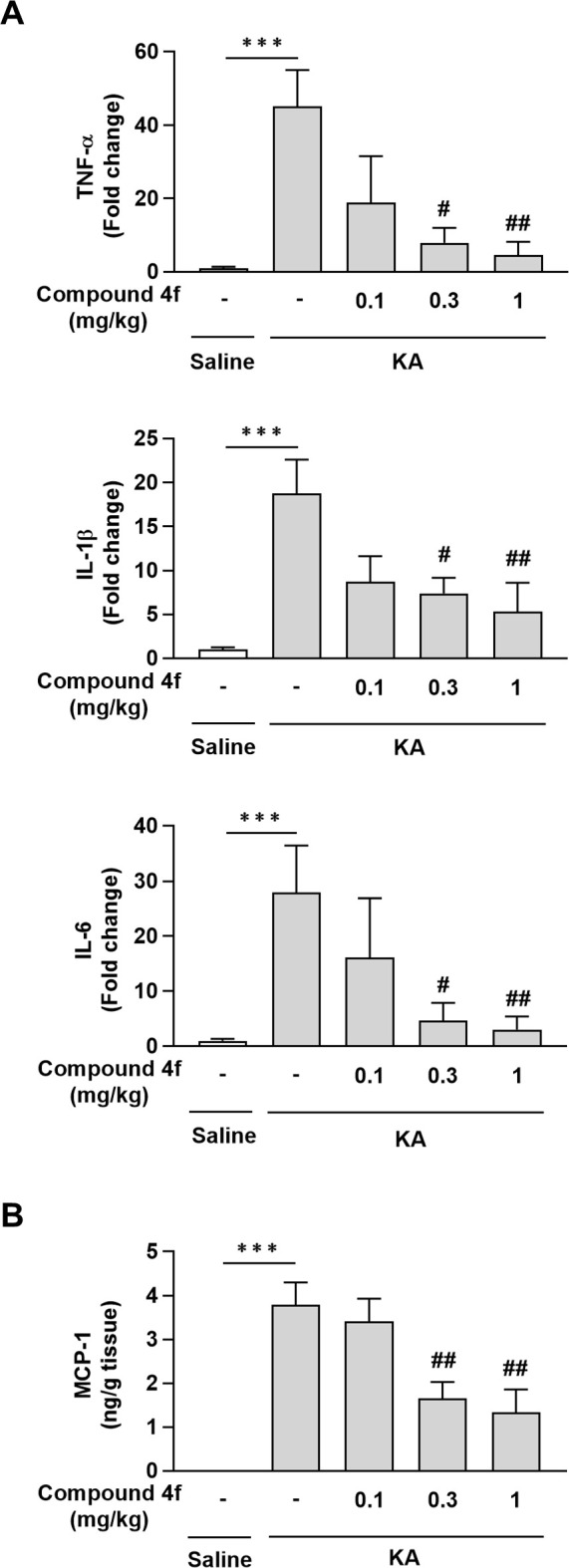
Effects of compound 4f on neuroinflammation in mice with KA-induced neurodegeneration. (A) Effects of compound 4f on the gene expression levels of tumor necrosis factor (TNF)-α, interleukin (IL)-1β, and IL-6 in the hippocampi of KA-treated mice. Mean ± standard error of the mean (SEM; n = 4 saline-injected mice; n = 8 KA-injected mice). ***P ≤ 0.001, compared to saline-injected mice (Student’s *t*-test). #*P* ≤ 0.01, ##*P* ≤ 0.001 compared to KA-injected mice treated with vehicle (Williams’ test). (B) Effects of compound **4f** on the expression levels of the monocyte chemotactic protein 1 (MCP-1) in the hippocampi of KA-treated mice. Results are presented as the mean ± SEM (n = 4 saline-injected mice; n = 8 KA-injected mice). ***P ≤ 0.001 compared to saline-injected mice (Aspin–Welch test). ##*P* ≤ 0.001 compared to KA-injected mice treated with vehicle (Williams’ test). IL, interleukin; KA, kainic acid; MCP-1, monocyte chemotactic protein-1; SEM, standard error of the mean; TNF, tumor necrosis factor.

KA treatment causes neuronal cell loss induced by excitotoxicity and neuroinflammation [21, 22]. First, we confirmed that compound **4f** decreased the seizure-like response elicited by KA (S1 Fig). Next, the effects of compound **4f** on neuroinflammation were evaluated in mice exposed to an i.c.v. injection of KA. The expression levels of TNF-α, IL-1β, and IL-6 mRNA (Fig 2A), as well as MCP-1 protein (Fig 2B), were significantly increased in the hippocampi of mice treated with KA compared to those in the saline-treated group. Compound **4f** at 0.3 and 1 mg/kg (once a day for 3 days) significantly decreased the levels of these inflammatory cytokines and chemokines induced by KA (*P* ≤ 0.01, Williams’ test; Fig 2A and 2B). Immunohistochemistry using anti-Iba-1 and GFAP antibodies also confirmed anti-neuroinflammatory effects of compound **4f** (S2 Fig). In saline-treated mice, GFAP-positive cells were small and their processes were long and thin. On the other hand, astrocyte cell bodies were enlarged, and their processes were shorter and thicker by KA injection, suggesting astrocyte activation. Cell bodies of Iba-1-positive cells in the hippocampus were also enlarged and cytoplasmic processes were thicker and shorter, displaying microglia activation whilst Iba-1-positive cells were ramified in saline-treated mice. The enlargement of astrocytes and microglia was suppressed by compound 4f. These results suggest that the activations of microglia and astrocytes in the hippocampus of KA-administered animals were suppressed by compound **4f**.

### Effects of compound 4f on neuronal cell loss in mice with KA-induced neurodegeneration

**Fig 3:**
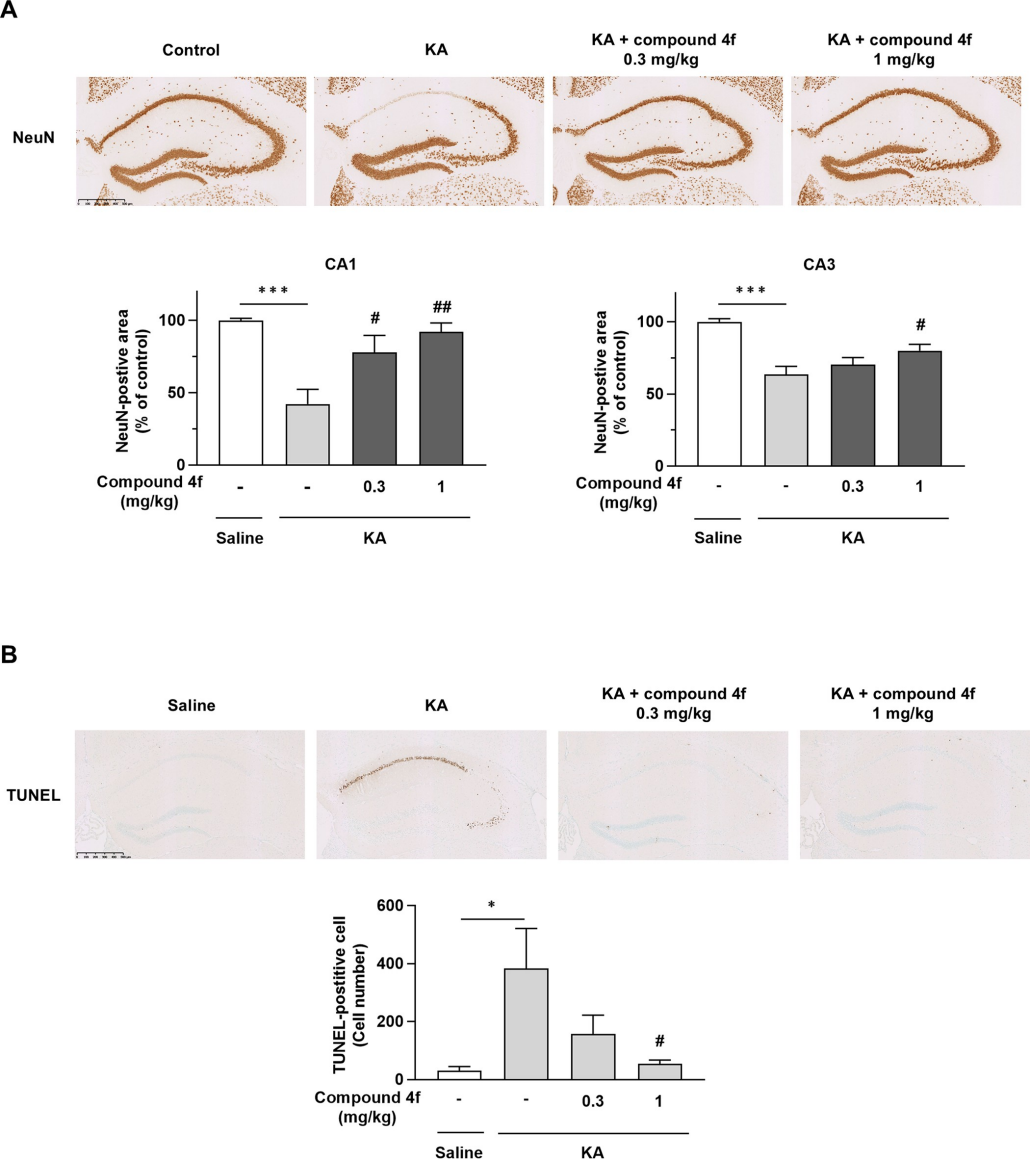
Effects of compound 4f on neuronal cell loss in mice with KA-induced neurodegeneration. (A) Effects of compound **4f** on neuronal cell loss in the hippocampus determined via immunohistochemistry using mAb to NeuN as a neuronal marker in mice. Upper, representative images of NeuN immunoreactivity. Scale bar = 500 μm. Lower, NeuN-positive area in CA1 and CA3 regions of the murine brain. Results are presented as the mean ± SEM, n = 9. ***P ≤ 0.001, compared to saline-injected mice (Aspin–Welch test). #*P* ≤ 0.05, ##*P* ≤ 0.01 compared to KA-injected mice treated with vehicle (Williams’ test). (B) Effects of compound **4f** on neuronal cell loss in the hippocampus determined via terminal deoxynucleotidyl transferase-mediated dUTP nick end-labeling (TUNEL) assay. Immunohistochemistry was performed using an *in situ* TUNEL staining kit. Upper, Representative images of TUNEL reactivity. Scale bar = 500 μm. Lower, numbers of TUNEL-positive cells in the murine hippocampus. Mean ± SEM, n = 9. *P ≤ 0.05 compared to saline-injected mice (Aspin-Welch test). #*P* ≤ 0.05 compared to KA-injected mice treated with vehicle (Shirley-Williams’ test). KA, kainic acid; SEM, standard error of the mean; TUNEL, terminal deoxynucleotidyl transferase-mediated dUTP nick-end labeling.

Next, we evaluated the number of NeuN-positive cells in the hippocampi of mice exposed to an i.c.v. injection of KA. The number of NeuN-positive cells was significantly decreased in the CA1 and CA3 regions of KA-injected mice compared to saline-injected mice (*P* ≤ 0.001, Aspin-Welch test; Fig 3A). Compound **4f** at 1 mg/kg (once a day for 3 days) significantly increased the number of NeuN-positive cells in KA-injected mice (*P* ≤ 0.05, Williams’ test; Fig 3A). Furthermore, the number of TUNEL-positive cells in the hippocampus was significantly increased owing to the induction of apoptosis by KA (Fig 3B). Compound **4f** significantly decreased the number of TUNEL-positive cells in the hippocampi of KA-injected mice (*P* ≤ 0.05, Shirley-Williams’ test; Fig 3B).

### Effects of compound 4f on cognitive function in mice with KA-induced neurodegeneration

**Fig 4:**
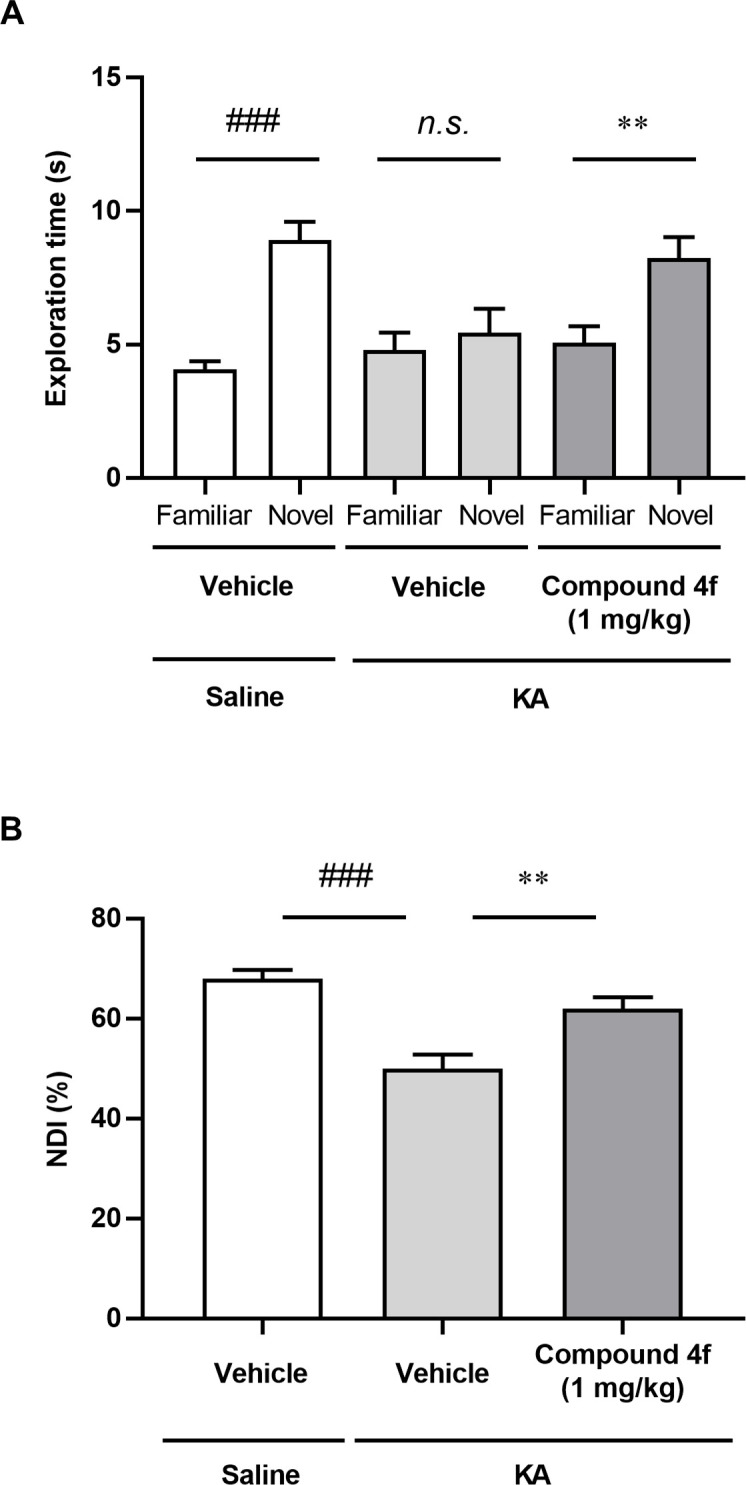
Effects of compound 4f on cognitive function in mice with KA-induced neurodegeneration. (A) Retention trial was performed 24 h after the acquisition trial, and the duration was measured. Results are presented as the mean ± SEM, n = 20 for vehicle-treated group of saline-injected mice. n = 28 for vehicle- and compound 4f-treated group of KA-injected mice. ###*P* ≤ 0.001 significant differences in exploration times for the novel object and the familiar object compared to saline-injected mice using the Aspin-Welch test. **P ≤ 0.01 significant differences in exploration times for the novel object and the familiar object in KA-injected mice treated with compound **4f** compared to the vehicle-treated group of KA-injected mice (Student’s *t*-test). (B) NDI was calculated based on the result of (A) as the ratio of the total time spent exploring the novel object to the total time spent exploring both objects multiplied by 100. Results are presented as the mean ± SEM, n = 20 for vehicle-treated group of saline-injected mice. n = 28 for vehicle- and compound 4f-treated group of KA-injected mice. ###*P* ≤ 0.001 compared to saline-injected mice using the Aspin-Welch test. **P ≤ 0.01 compared to KA-injected mice treated with vehicle (Student’s *t*-test). KA, kainic acid; *n*.*s*.; not significant; NDI, novelty discrimination index; SEM, standard error of the mean.

KA-induced lesions in the hippocampal CA1 and CA3 regions result in cognitive impairment [28]. We investigated whether compound **4f** improved cognitive impairment in KA-injected mice by assessing their performance in NORT. We confirmed that the exploration time for the novel object was significantly longer than that for the familiar object in saline-injected mice in the retention trial, whereas no significant difference was observed in the exploration time for the novel and the familiar objects in KA-injected mice (*P* ≤ 0.001, Aspin-Welch test; Fig 4A). Moreover, KA-injected mice showed a significant decrease in NDI in the retention trial compared with saline-injected mice (Fig 4B). Treatment with compound **4f** at 1 mg/kg increased the exploration time for the novel object and NDI compared to vehicle treatment in KA-injected mice (*P* ≤ 0.01, Student’s t-test; Fig 4A and 4B).

### Effects of cannabinoid receptor antagonists on compound 4f-mediated neuroprotection in mice with KA-induced neurodegeneration

**Fig 5:**
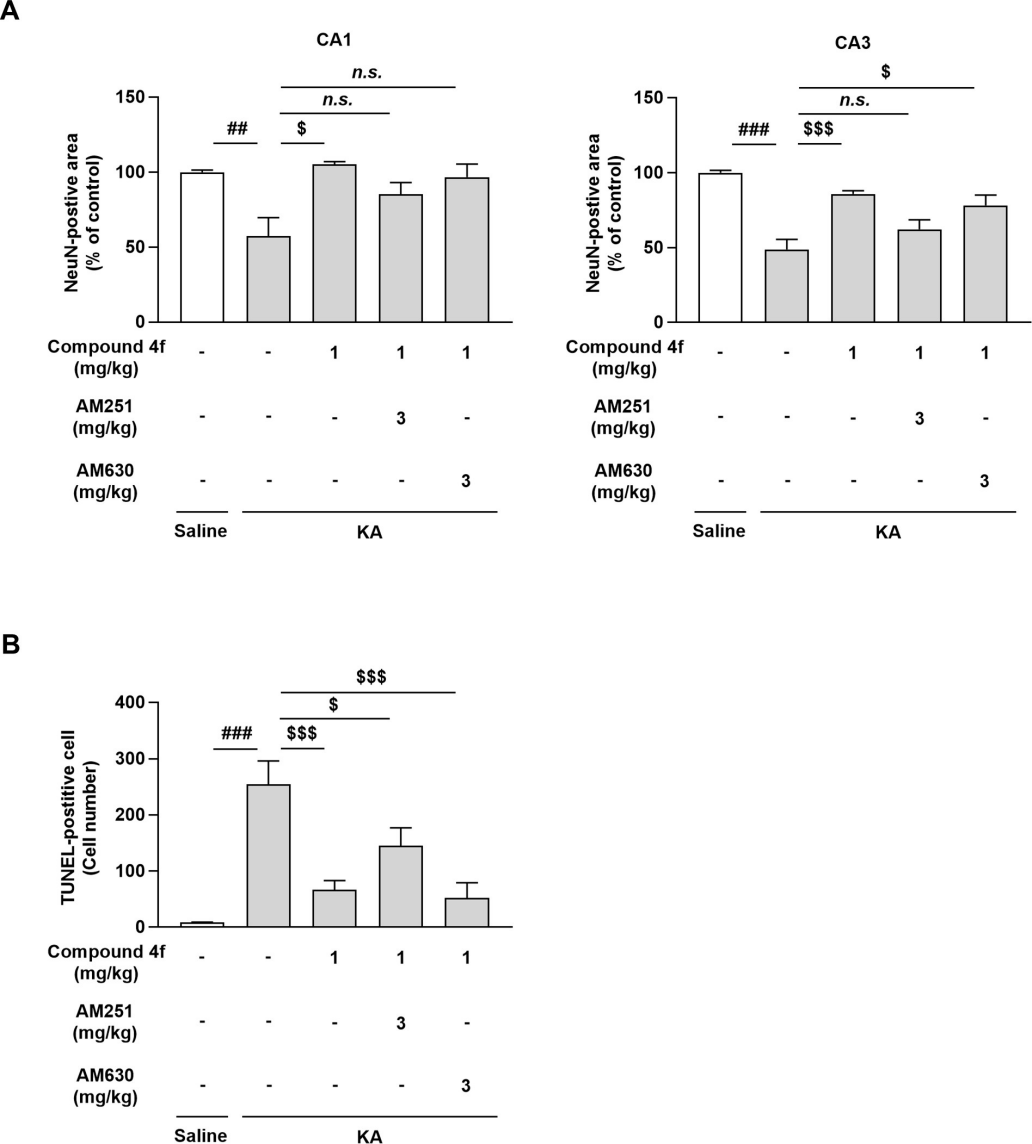
The effects of cannabinoid receptor antagonists against neuroprotective effects of compound 4f in KA-treated mice. (A) Immunohistochemistry was performed with an anti-NeuN antibody to assess NeuN in the hippocampus. NeuN-positive areas in CA3 or CA1 regions of the murine brain. Mean ± SEM (n = 4 for saline-injected mice; n = 8 for vehicle-treated group of KA-injected mice, n = 9 for drugs-treated groups of KA-injected mice). ##*P* ≤ 0.01, ###*P* ≤ 0.001 compared to saline-injected mice (Aspin-Welch test). $*P* ≤ 0.05, $ $ $*P* ≤ 0.001 compared to KA-injected mice treated with vehicle (Steel’s multiple comparison test). (B) Immunohistochemistry was performed using an *in situ* TUNEL staining kit. Lower, numbers of TUNEL-positive cells in the murine hippocampus. Mean ± SEM (n = 4 for saline-injected mice; n = 8 for vehicle-treated group of KA-injected mice, n = 9 for drugs-treated groups of KA-injected mice). ###*P* ≤ 0.001 compared to saline-injected mice (Aspin-Welch test). $*P* ≤ 0.05, $ $ $*P* ≤ 0.001 compared to KA-injected mice treated with vehicle (Dunnett’s multiple comparison test). KA, kainic acid; AM251, CB1R antagonist; AM630, CB2R antagonist; *n*.*s*.; not significant; SEM, standard error of the mean; TUNEL, terminal deoxynucleotidyl transferase-mediated dUTP nick-end labeling.

To clarify the involvement of cannabinoid receptor activation in the neuroprotective effects of compound **4f**, we administered the CB1R antagonist AM251 or CB2 antagonist AM630 to KA-injected mice before compound **4f** administration and evaluated neuronal cell loss by assessing the numbers of NeuN-positive and TUNEL-positive cells. Compound **4f** significantly prevented the reduction in NeuN-positive cells in the CA1 of KA-injected mice, whereas the combination of compound **4f** and AM251 or AM630 did not show significant differences compared to the KA-treated group (*P* ≤ 0.01, Steel’s multiple comparison test; Fig 5A and S3 Fig). Compound **4f** also significantly prevented the reduction of NeuN-positive cells in the CA3 of KA-injected mice, and the combination of compound **4f** with AM251 did not significantly prevent the reduction in NeuN-positive cells in the CA3 region, whereas its combination with AM630 still led to significant neuroprotective effects of compound **4f** (*P* ≤ 0.01, Steel’s multiple comparison test; Fig 5A). Moreover, the combination of compound **4f** with AM251 or AM630 still showed significant effects in the TUNEL assay (*P* ≤ 0.01, Dunnett’s multiple comparison test; Fig 5B and S3 Fig). These results indicate that the effects of compound **4f** partly involve the activation of cannabinoid signaling, mainly via CB1R.

### Gene expression profile and pathway analysis to elucidate the mechanism of action of compound 4f

**Fig 6:**
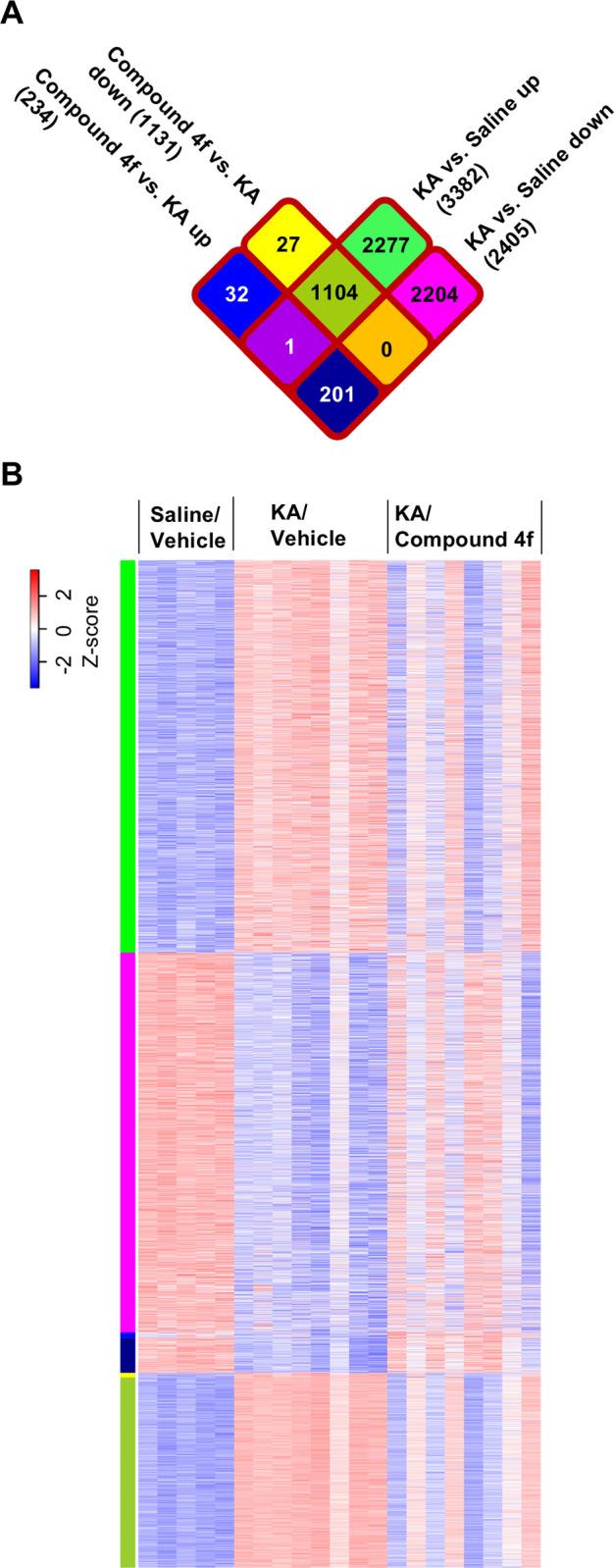
Comparison of altered gene expression across groups of saline/vehicle-, KA/vehicle-, and KA/compound 4f-treated mice. (A) Venn diagram showing the number of genes showing fold change (FC) ≥ 1.2 and false discovery rate < 0.05 among all groups of treated mice. KA, kainic acid. (B) Heatmap showing the gene expression changes (blue indicates FC ≤ −1.2; red indicates FC ≥ 1.2) in each mouse in saline/vehicle-, KA/vehicle-, and KA/compound **4f**-treated groups. Colors in the left of heat map are corresponding to those in the above-mentioned Venn diagram.

To understand the effects of compound **4f** on the pathophysiological pathways involved in KA-induced neurodegeneration, global expression changes were evaluated in the hippocampi of mice across three groups (saline-injected mice, KA-injected mice treated with vehicle, and KA-injected mice treated with compound **4f**) using RNA sequencing (RNA-seq). RNA-seq libraries were generated using the same brain tissue samples used for qPCRs (Fig 2) and were loaded onto an Illumina HiSeq platform. Various genes exhibited altered expression with a fold change ≥ 1.2 and false discovery rate < 0.05, among the three groups (Fig 6A). In total, 3382 and 2204 DEGs were upregulated and downregulated, respectively, following KA injection compared with saline-injected mice. Among the DEGs in the KA-injected mice, compound **4f** reversed the expression change of 1104 upregulated and 201 downregulated DEGs, indicating that compound **4f** partially rescued the KA-induced pathophysiological changes in gene expression. The DEGs of each mouse in the three groups are displayed as a heatmap (Fig 6B). The pattern of DEGs in KA-injected mice was distinct from that in saline-injected mice. This pattern in KA-injected mice was changed by the treatment of compound **4f**, with the tendency of rectification to the pattern in saline-injected mice, although there were some inter-individual variations. Among the DEGs, the expression levels of CB1R, CB2R, and MAGL were significantly altered in the hippocampi of mice treated with KA compared with those in the saline-injected group (S4 Fig). The increased CB2R expression in KA-injected mice was significantly reversed by compound **4f** treatment, while CB1R and MAGL expression did not change.

To identify the signaling pathways rescued by compound **4f**, global expression data were analyzed using the Ingenuity Pathway Analysis (IPA). Several immune response pathways were listed among the top 20 signaling pathways identified as DEGs that were upregulated in KA-induced neurodegeneration (Table 1). Several neurotransmitter and other nervous system signaling pathways were included in the top 20 signaling pathways specified for downregulated DEGs (Table 2). Treatment with compound **4f** partly reversed the expression of genes that were upregulated or downregulated by KA injection (Tables 3 and 4); the KA-injected mice treated with compound **4f** showed downregulation of some of the immune response pathways induced by KA and upregulation of some of the neurotransmitter and other nervous signaling pathways suppressed by KA. The cannabinoid neuronal synapse pathway, which was downregulated by KA, was not included in the top 20 pathways altered in KA-injected mice treated with compound **4f**. The ratio of genes reversely changed by compound **4f** to genes changed by KA was relatively high in the top 20 pathways (Tables 3 and 4), such as 60 to 99 in neuroinflammation, 9 to 39 in γ-aminobutyric acid (GABA), and 5 to 27 in glutamate signaling pathways. In contrast, it was low in pathways, such as endocannabinoid neuronal synapses, with a ratio of 5 to 48 (S2 Table). Taken together, these results indicate that compound **4f** regulates the neuroinflammatory and neurotransmitter pathways in KA-induced neurodegeneration.

**Table 1:** Top 20 Ingenuity Pathway Analysis (IPA) pathways of 3382 genes upregulated following KA treatment with a *P*-value of < 0.05.

Pathways	-log (*P* value)	Rank
Hepatic fibrosis signaling pathway	23.400	1
Hepatic fibrosis/Hepatic stellate cell activation	21.800	2
**Systemic lupus erythematosus in B-cell signaling pathway**	20.500	3
Role of macrophages, fibroblasts, and endothelial cells in rheumatoid arthritis	19.900	4
Molecular mechanisms of cancer	19.900	5
Coronavirus pathogenesis pathway	18.100	6
**Phagosome formation**	17.100	7
**Neuroinflammation signaling pathway**	16.400	8
Tec kinase signaling	16.200	9
Osteoarthritis pathway	16.000	10
**IL-8 signaling**	15.800	11
**Dendritic cell maturation**	14.000	12
Colorectal cancer metastasis signaling	13.600	13
**NF-κB signaling**	13.500	14
**Role of pattern recognition receptors in the recognition of bacteria and viruses**	13.000	15
**Leukocyte extravasation signaling**	12.900	16
STAT3 pathway	12.700	17
**TREM1 signaling**	12.700	18
Kinetochore metaphase signaling pathway	12.600	19
Cardiac hypertrophy signaling (enhanced)	12.600	20

Bold: cellular immune response pathways

KA, kainic acid; IPA, Ingenuity Pathway Analysis

**Table 2:** Top 20 IPA pathways of 2405 genes downregulated following KA treatment with a *P*-value of < 0.05.

Pathways	-log (*P* value)	Rank
**Endocannabinoid neuronal synapse pathway**	18.300	1
**Opioid signaling pathway**	17.100	2
**GABA receptor signaling**	16.600	3
**Synaptogenesis signaling pathway**	16.300	4
Calcium signaling	15.200	5
**Glutamate receptor signaling**	13.500	6
**Synaptic long-term depression**	12.700	7
Role of NFAT in cardiac hypertrophy	12.100	8
**Netrin signaling**	11.800	9
nNOS signaling in skeletal muscle cells	11.500	10
CCR5 signaling in macrophages	11.100	11
Corticotropin releasing hormone signaling	10.400	12
G beta-gamma signaling	10.300	13
**GPCR-mediated nutrient sensing in enteroendocrine cells**	10.200	14
**GNRH signaling**	10.100	15
**nNOS signaling in neurons**	10.100	16
**CREB signaling in neurons**	9.280	17
Androgen signaling	8.890	18
**Dopamine-DARPP32 feedback in cAMP signaling**	8.740	19
**Synaptic long-term potentiation**	7.810	20

Bold: neurotransmitter and other nervous system signaling pathways

KA, kainic acid; IPA, Ingenuity Pathway Analysis

**Table 3:** Top 20 IPA pathways of 1131 genes downregulated by compound 4f among genes upregulated following KA treatment with a *P*-value of < 0.05.

Pathways	-log (*P* value)	Rank
**Systemic lupus erythematosus in B-cell signaling pathway**	21.000	1
**Neuroinflammation signaling pathway**	20.500	2
**Phagosome formation**	19.300	3
Role of hypercytokinemia/hyperchemokinemia in the pathogenesis of influenza	18.100	4
**Role of pattern recognition receptors in the recognition of bacteria and viruses**	16.000	5
Hepatic fibrosis signaling pathway	14.900	6
**TREM1 signaling**	13.600	7
Tec kinase signaling	12.800	8
**Antigen presentation pathway**	12.400	9
Integrin signaling	12.000	10
Regulation of cellular mechanics by calpain protease	11.800	11
**IL-4 signaling**	11.400	12
**Dendritic cell maturation**	11.300	13
**T-helper cell differentiation**	11.000	14
Glioma invasiveness signaling	11.000	15
**Th1 and Th2 activation pathway**	10.800	16
**Leukocyte extravasation signaling**	10.700	17
**Agranulocyte adhesion and diapedesis**	10.700	18
**T-cell exhaustion signaling pathway**	10.500	19
**NF-κB signaling**	10.300	20

Bold: cellular immune response pathways

KA, kainic acid; IPA, Ingenuity Pathway Analysis

**Table 4:** Top 20 IPA pathways of 234 genes upregulated by compound 4f among genes downregulated following KA treatment with a *P*-value of < 0.05.

Pathways	-log (*P* value)	Rank
**GABA receptor signaling**	6.730	1
**CREB signaling in neurons**	5.130	2
Gap junction signaling	4.910	3
Glutamate-dependent acid resistance	4.100	4
Calcium signaling	3.980	5
**Synaptogenesis signaling pathway**	3.910	6
**Glutamate receptor signaling**	3.810	7
**Synaptic long-term depression**	3.510	8
**Serotonin receptor signaling**	3.230	9
Glutamate degradation III (via 4-aminobutyrate)	3.110	10
G-Protein coupled receptor signaling	3.080	11
Breast cancer regulation by stathmin1	3.040	12
Chondroitin sulfate biosynthesis	2.800	13
**Opioid signaling pathway**	2.750	14
Dermatan sulfate biosynthesis	2.720	15
Corticotropin-releasing hormone signaling	2.710	16
**Neuropathic pain signaling in dorsal horn neurons**	2.670	17
cAMP-mediated signaling	2.330	18
Antiproliferative role of somatostatin receptor 2	2.300	19
Gαi signaling	2.270	20

Bold: neurotransmitter and other nervous system signaling pathways

KA, kainic acid; IPA, Ingenuity Pathway Analysis

## Discussion

Here, we demonstrated for the first time that compound **4f**, a selective reversible MAGL inhibitor, exerted neuroprotective effects by ameliorating neuroinflammation in KA-injected mice. A single oral administration of compound **4f** showed rapid brain penetration and induced robust elevation of 2-AG levels in a dose-dependent manner, and the pharmacokinetics of compound **4f** and pharmacodynamics of 2-AG were well correlated (Fig 1B and 1C). To investigate the TO of compound **4f** on MAGL in the brain, we used our previously reported non-labeled MAGL tracer, T-211 [23]. T-211 was highly accumulated in the cortices and hippocampi of mice, and the binding of T-211 was almost completely abolished when it was administered to MAGL-knockout mice, indicating the specific binding of T-211 to MAGL in the brain [23]. In the present study, brain Kp values of T-211 were used to estimate the TO to normalize the brain concentrations to paired plasma concentrations. T-211 accumulation in the cortex and hippocampus was displaced by compound 4f administration in a dose-dependent manner (Fig 1D). The TO of compound **4f** after treatment at 10 mg/kg was set at 100%, where 2-AG/AA changes reached the plateau phase, and the brain Kp values of T-211 exhibited an asymptotic decrease for compound **4f** doses of up to 10 mg/kg. No significant increase in 2-AG levels was observed in the brain when compound **4f** was administered to mice at 0.1 mg/kg with a TO of 50%. In contrast, a significant increase in 2-AG levels was observed with compound **4f** at 1 mg/kg, which exhibited almost 100% MAGL occupancy (Fig 1D). This result was consistent with a previous result that no increase in 2-AG levels is observed in the brains of MAGL heterozygous knockout mice [29]. These results indicate that compound **4f** is a potent brain-penetrant MAGL inhibitor. Furthermore, we reported a ligand to MAGL for positron emission tomography imaging studies in rodents and non-human primates [23]. Future investigations of TO studies using this translational research tool can aid in more precise predictions of the clinical dose regimen.

Compound **4f** prevented neuroinflammation, neuronal cell loss, and cognitive impairment in a mouse model of KA-induced neurodegeneration (Figs 2–4 and S2 Fig). Two mechanisms of action can be hypothesized for the anti-inflammatory and neuroprotective effects of this MAGL inhibitor: (1) accumulated 2-AG can activate the cannabinoid receptor or (2) AA and eicosanoid reduction can repress inflammation in the brain. 2-AG is an endocannabinoid and endogenous agonist of cannabinoid receptors. AA and its metabolites, including prostaglandins, can increase inflammatory effects via prostaglandin receptor signaling. In this study, the CB1R antagonist AM251 and the CB2R antagonist AM630 partly prevented the neuroprotective effects of compound **4f** (Fig 5). The inhibitory levels of these antagonists on the effect of compound **4f** in NeuN and the TUNEL assay were different, which might be affected by the statistical power based on the change in the numbers of NeuN- or TUNEL-positive cells after KA treatment compared with saline treatment.

2-AG is a full agonist of CB1R and CB2R, with equal agonistic properties. CB1R is highly expressed in neurons, and the endocannabinoid system plays a role in balancing neuronal excitability by activating CB1R. In a mouse model of traumatic brain injury, 2-AG attenuated edema formation via CB1R activation and exerted neuroprotective effects [11]. KA-mediated seizure susceptibility and vulnerability of neurons were increased by genetic deletion of CB1Rs, and inactivation of CB1 signaling by a CB1R antagonist decreased the threshold for KA-induced seizures [30]. Additionally, a CB2R selective agonist attenuated neuroinflammation and neurovascular injury [31]. These studies support our results that the neuroprotective effects of MAGL inhibition are attributed to CB1 and CB2 signaling via 2-AG.

The inhibition of AA cascade signaling by MAGL inhibitors may also be involved in the neuroprotective and anti-inflammatory effects. A recent study showed that an irreversible MAGL inhibitor reduced benzodiazepine-refractory status epilepticus and prevented cell loss and cognitive deficits induced by KA injection into the amygdala [32]. The authors demonstrated that these effects of MAGL inhibitor were also observed in CB1R-deficient mice, suggesting that the effects of MAGL inhibition may be attributed to a reduction in AA rather than CB1 signaling via increased 2-AG levels. The experimental conditions, such as the timing of treatment with MAGL inhibitors, were not identical to those in our study. Mice in the study by Terrone et al. [32] were treated with the irreversible MAGL inhibitor CPD-4645 after status epilepticus onset (KA treatment), whereas mice were treated with the MAGL inhibitor compound **4f** prior to KA treatment in our study. Moreover, Gobbo and O’Mara [33] showed that post-treatment, but not pre-treatment, with the selective cyclooxygenase-2 (COX-2) inhibitor celecoxib improved performance in spatial and non-spatial tasks in a model of KA-induced neurodegeneration compared to the vehicle-treated group. These results indicated that the effects of post-treatment with the MAGL inhibitor could be attributed to AA reduction in the KA-treated model. These findings suggest that both AA reduction and 2-AG increase might contribute to the efficacy of MAGL inhibition on neuroinflammation, neuronal loss, and subsequent cognitive impairment induced by KA via different mechanisms. Elevated 2-AG levels mainly contribute to the enhancement of endocannabinoid signaling, and AA reduction contributes to the anti-neuroinflammatory response under conditions of KA treatment.

Global expression analysis using RNA-seq was used to define the transcriptional signature in the hippocampi of mice across three groups: saline-injected mice, KA-injected mice treated with vehicle, and KA-injected mice treated with compound **4f**. Venn figure and heat map analysis showed different patterns of gene expression between saline-treated and KA-treated mice, and the pattern of compound **4f**-treated mice was similar to that in saline-treated mice, with some individual variation. IPA revealed upregulated immune response pathways and downregulated neurotransmitter and other nervous system signaling pathways induced by KA (Tables 1 and 2). These results indicate that neuroinflammation and neuronal dysfunction are involved in KA-induced neurodegeneration and cognitive impairment, which is consistent with the results of previous studies [34]. Treatment with compound **4f** partially restored these gene clusters to the saline-treated levels. Compound **4f** downregulated immune response pathways, which was consistent with the results of qPCR (Fig 2). Furthermore, compound **4f** upregulated neurotransmitters and other nervous system signaling pathways, reflecting the prevention of neuronal loss and subsequent cognitive impairment (Tables 3 and 4). Compound **4f** altered the DEGs induced by KA treatment in the neuroinflammation, GABA, and glutamate signaling pathways (S2 Table). It is reasonable that compound **4f** showed partial but not complete rescue of the signaling pathways, as compounds **4f**, 2-AG, and AA are not direct KA receptor modulators. Interestingly, the expression of genes related to the endocannabinoid neuronal synapse pathway, which can be modulated by 2-AG produced by MAGL inhibition, was not changed by compound **4f** in mice treated with KA, whereas the CB receptor antagonists AM251 and AM630 partly prevented the compound **4f** effects (Fig 5). The limited effects of compound **4f** may be owing to the significant decrease in CB1R expression by KA treatment in the hippocampi of KA-injected mice (S4 Fig). The impact of compound **4f** on gene expression changes in the CB signaling pathways should be further investigated in accordance with the time course of changes in 2-AG levels by the compound. The altered IPA pathways from upregulated or downregulated genes after treatment with KA and compound **4f** in this study reflect the status of the hippocampus 24 h after the last administration of compound **4f**, where 2-AG was restored to the baseline level according to the time course analysis in Fig 1C. Time-course analysis of gene expression related to CB signaling pathways would also provide further understanding of the difference in the type of inhibition of MAGL by treatment with compound **4f**, an irreversible-type MAGL inhibitor, or other cannabinoid agonists and antagonists.

We demonstrated that compound **4f** significantly attenuated MCP-1 expression, which was enhanced by the i.c.v. injection of KA (Fig 2B). MCP-1 upregulation by KA treatment in the hippocampus plays an important role in disrupting the blood-brain barrier (BBB) and peripheral immune cell recruitment into the brain [35]. It has also been reported that serum MCP-1 levels are increased in mild cognitive impairment and mild AD [36]. These findings indicate a link between BBB disruption, neuroinflammation, and neurodegenerative disease. More recently, pharmacological inhibition of MAGL with CPD-4645 was shown to improve BBB breakdown after chronic lipopolysaccharide administration [37]. Further experiments will be needed to understand the effects of MAGL inhibitors on BBB-protective, anti-inflammatory, and neuroprotective effects.

These fascinating properties of MAGL inhibition may have advantages over anti-inflammatory agents, including COX-2 inhibitors, as MAGL inhibition could be more effective in chronic neurodegenerative diseases in which hyperexcitability and neuroinflammation occur during disease progression via at least two pathways modulated by increased 2-AG and decreased AA.

Although we did not examine the effects of compound **4f** on KA-induced hyperexcitability in the murine brain, previous reports have suggested that the effects of endocannabinoid signal modulation and MAGL inhibition on hyperexcitability are controversial because of the complexities of endocannabinoid signaling and the use of different animal models or drugs (summarized in [38]). Additionally, it has been suggested that cannabinoid signaling modulation could have both therapeutic benefits and pro-epileptic risks depending on the way of modulation in which receptor desensitization might be involved. Compound **4f**, a reversible MAGL inhibitor, induced a transient increase in 2-AG in correlation with the compound pharmacokinetics, which would be a favorable profile to lower the risk of desensitization of CB1R and CB2R than irreversible MAGL inhibitors, which induce prolonged 2-AG accumulation. Therefore, a reversible MAGL inhibitor can be a good tool for clarifying the therapeutic potential of MAGL inhibition on hyperexcitability.

Reversible inhibitors also have advantages over irreversible inhibitors in terms of safety [39]. Multiple doses of the irreversible MAGL inhibitor, Lu AG06466, failed to meet the primary endpoint for treating Tourette syndrome in a phase 2 study, although it reduced the tics and premonitory urges in patients with Tourette syndrome in a single-dose phase 1b study, suggesting good tolerability [40]. Since prolonged 2-AG accumulation via irreversible MAGL inhibition is suggested as one of the causes for safety issues, reversible inhibition has the potential to mitigate this issue and to bring beneficial effects of multiple doses in treatment.

## Supporting information

S1 Fig Effects of compound 4f on the seizure-like response elicited by kainic acid (KA). Various concentrations (0.1, 0.3, and 1 mg/kg) of compound 4f were administered to seven-week-old mice. One hour after compound 4f administration, the mice were anesthetized with pentobarbital sodium (50 mg/kg, intraperitoneal; Somnopentyl®, Kyoritsu Seiyaku, Tokyo, Japan) and fixed to a stereotaxic apparatus (Kopf Instruments, Tujunga, CA, USA). For intracerebroventricular (i.c.v.) administration of KA (0.2 μg), an injection cannula was implanted into the left lateral ventricle (0.2 mm posterior to bregma, 1.0 mm lateral to the midline, 2.0 mm depth from the skull surface). Seizure scores were measured for 5 min by a blinded observer 2 h after KA injection. Data are presented as the mean ± standard error of the mean (SEM; n = 5 for saline-injected mice; n = 7 for vehicle-treated group of KA-injected mice, n = 8 for compound 4f-treated groups of KA-injected mice). **P ≤ 0.01 compared to saline-injected mice (Wilcoxon test). #*P* ≤ 0.025 compared to KA-injected mice treated with vehicle (Shirley–Williams’ test). KA, kainic acid; SEM, standard error of the mean.
(TIF)

S2 Fig Immunohistochemistry using anti-Iba-1 and GFAP antibodies. Those antibodies’ information was described in S2 File. Scale bar = 500 μm for whole hippocampus, 50 μm for CA1 and CA3. Arrow head: activated cells.
(TIF)

S3 Fig Representative image data in Fig 5. Immunohistochemistry using an anti-NeuN antibody was performed to detect NeuN in the hippocampus. Immunohistochemistry using *in situ* terminal deoxynucleotidyl transferase-mediated dUTP nick end-labeling (TUNEL) staining was performed to assess the apoptotic cells. Scale bar = 500 μm.
(TIF)

S4 Fig Gene expression levels of *CB1R*, *CB2R*, and *MAGL* in KA-injected mice treated with compound 4f. Mean ± SEM (n = 5 for vehicle-treated group of saline-injected mice; n = 8 for vehicle- or compound 4f- treated groups of KA-injected mice). *P*-values were adjusted for multiple comparisons using the Benjamini–Hochberg false discovery rate. *Adjusted *P* value ≤ 0.05, compared to saline-injected mice or KA-injected mice.
(TIF)

S1 Table *In vitro* assay profile of compound 4f using the enzyme and binding assay. *In vitro* assay profile was performed by Eurofins Cerep PanLabs Taiwan, Ltd. (Taipei, Taiwan). The basic methods employed in this study were adapted from scientific literature to maximize the reliability and reproducibility. Reference standards were used as an integral part of each assay to ensure the validity of the results. Items meeting the criteria for significance (≥50% stimulation or inhibition) are highlighted. bov = bovine; gp = guinea pig; ham = hamster; hum = human; pig = porcine. %Inh.: % of inhibition. %Cont.: % of control.
(PDF)

S2 Table Compound 4f restores the expression of several genes changed by kainic acid (KA) in neuroinflammation, γ-aminobutyric acid (GABA) receptor, glutamate receptor signaling, and endocannabinoid neuronal synapse pathways. *P* value for KA-injected mice treated with compound 4f compared to KA-injected mice treated with the vehicle. Ratio (KA + vehicle/KA + compound **4f**): Number of genes significantly changed by compound **4f** among those genes changed by KA (FC ≤ -1.2 or ≥ 1.2)/number of genes changed by KA (FC ≤ -1.2 or ≥ 1.2).
(PDF)

S1 File Schematic diagram illustrating experimental design of KA-induced neurodegeneration model. (PDF)

S2 File Experimental procedures in supporting information. (PDF)

S3 File All data in the manuscript and supporting information. (XLSX)
