# Supplementary figures and images for: Compound 4f, a novel brain-penetrant reversible monoacylglycerol inhibitor, ameliorates neuroinflammation, neuronal cell loss, and cognitive impairment in mice with kainic acid-induced neurodegeneration
Source: PLoS One. 2024 Nov 21;19(11):e0312090. doi: 10.1371/journal.pone.0312090 (PMC11581214; doi:10.1371/journal.pone.0312090)

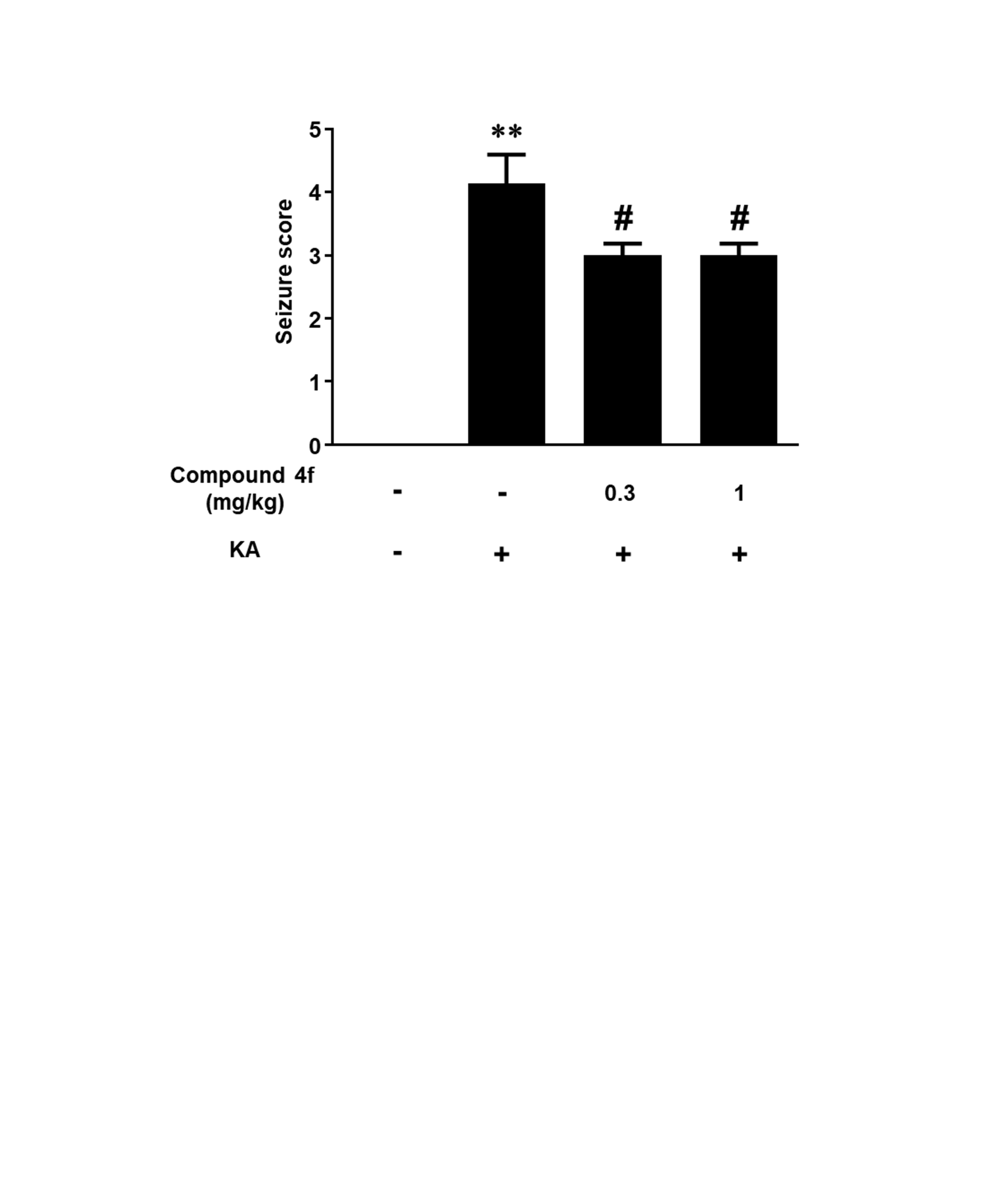

Supplement: S1 Fig — Various concentrations (0.1, 0.3, and 1 mg/kg) of compound 4f were administered to seven-week-old mice. One hour after compound 4f administration, the mice were anesthetized with pentobarbital sodium (50 mg/kg, intraperitoneal; Somnopentyl®, Kyoritsu Seiyaku, Tokyo, Japan) and fixed to a stereotaxic apparatus (Kopf Instruments, Tujunga, CA, USA). For intracerebroventricular (i.c.v.) administration of KA (0.2 μg), an injection cannula was implanted into the left lateral ventricle (0.2 mm posterior to bregma, 1.0 mm lateral to the midline, 2.0 mm depth from the skull surface). Seizure scores were measured for 5 min by a blinded observer 2 h after KA injection. Data are presented as the mean ± standard error of the mean (SEM; n = 5 for saline-injected mice; n = 7 for vehicle-treated group of KA-injected mice, n = 8 for compound 4f-treated groups of KA-injected mice). **P ≤ 0.01 compared to saline-injected mice (Wilcoxon test). #P ≤ 0.025 compared to KA-injected mice treated with vehicle (Shirley–Williams’ test). KA, kainic acid; SEM, standard error of the mean. (TIF) [file pone.0312090.s001.tif]

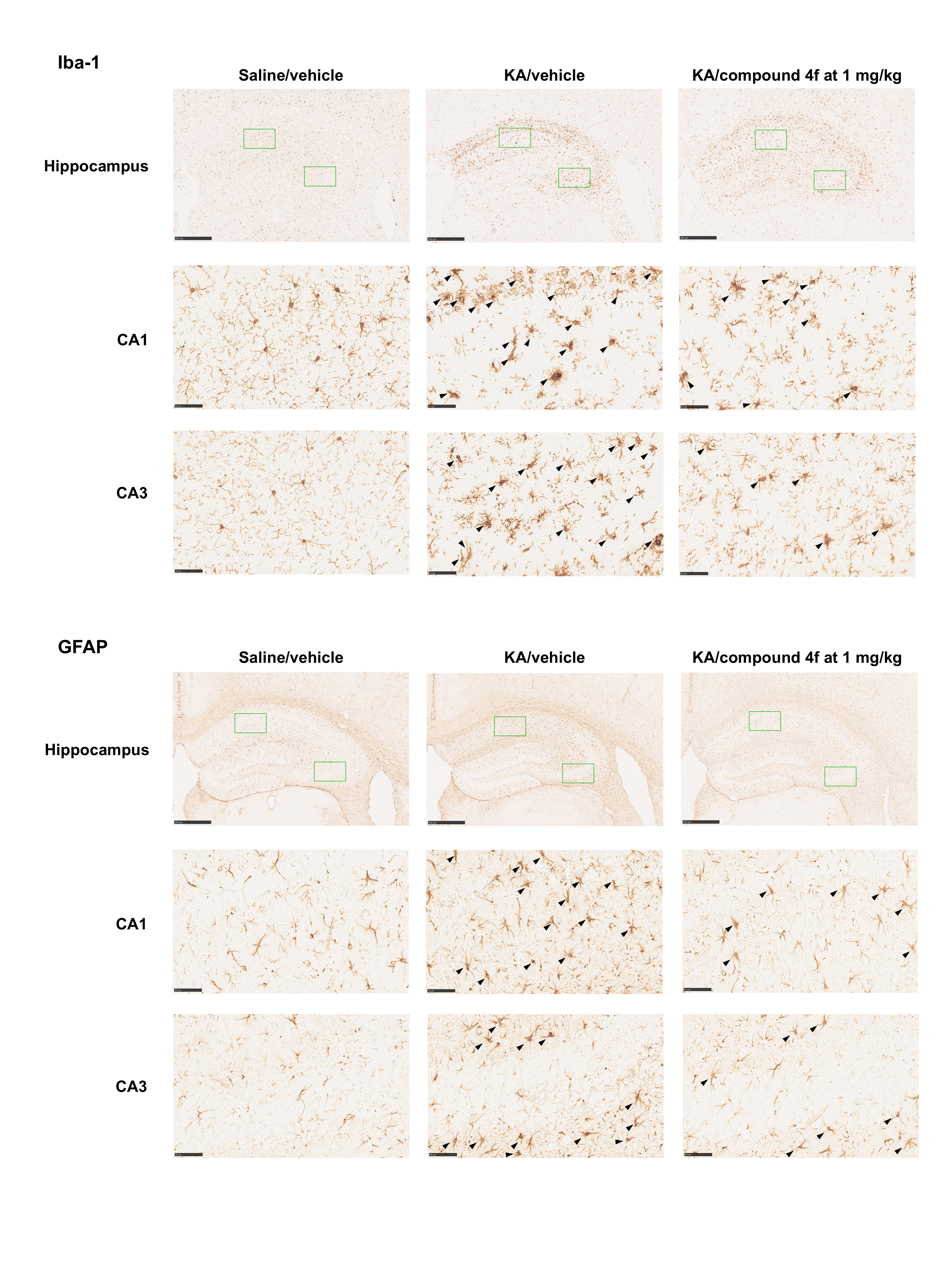

Supplement: S2 Fig — Those antibodies’ information was described in S2 File. Scale bar = 500 μm for whole hippocampus, 50 μm for CA1 and CA3. Arrow head: activated cells. (TIF) [file pone.0312090.s002.tif]

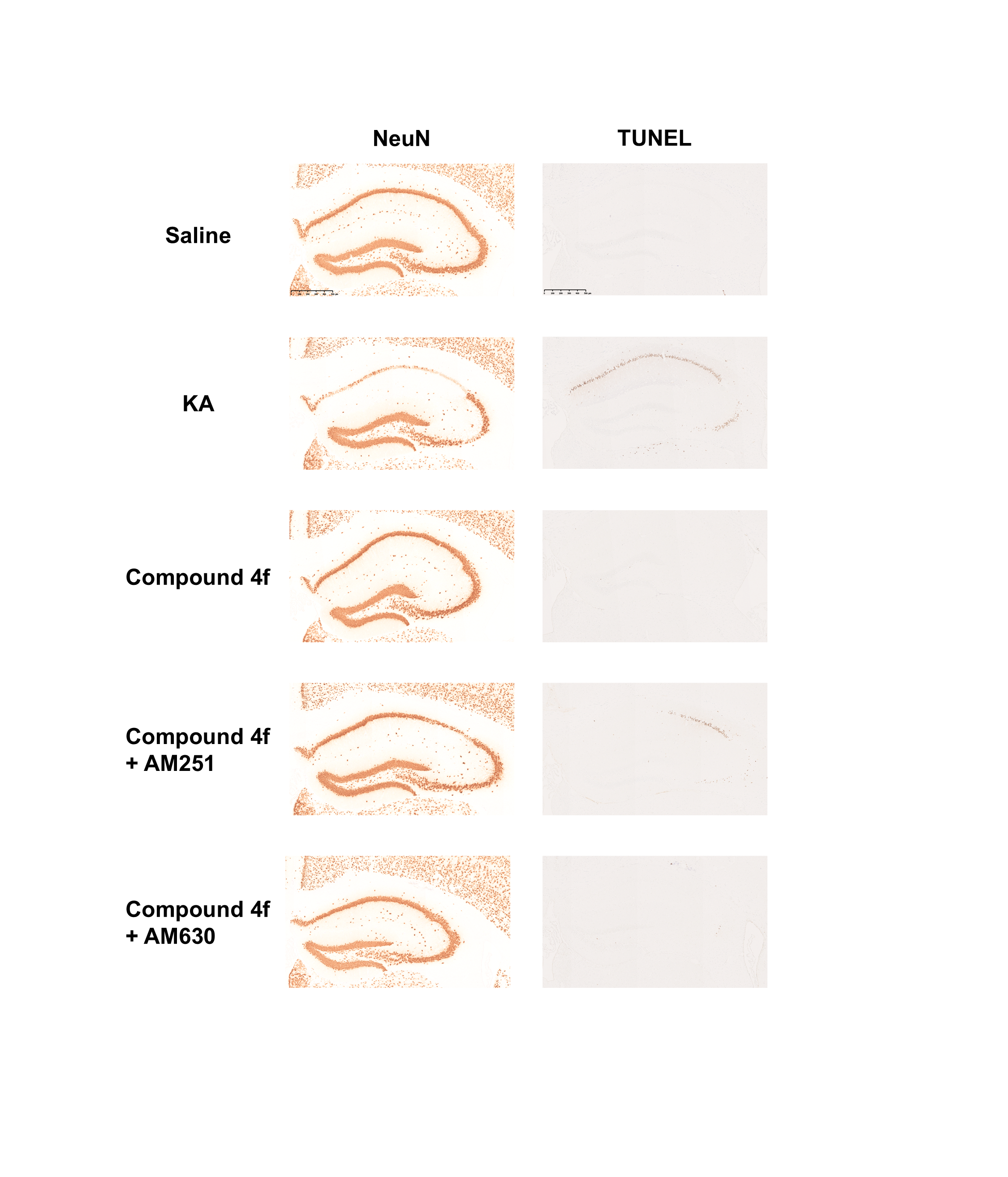

Supplement: S3 Fig — Immunohistochemistry using an anti-NeuN antibody was performed to detect NeuN in the hippocampus. Immunohistochemistry using in situ terminal deoxynucleotidyl transferase-mediated dUTP nick end-labeling (TUNEL) staining was performed to assess the apoptotic cells. Scale bar = 500 μm. (TIF) [file pone.0312090.s003.tif]

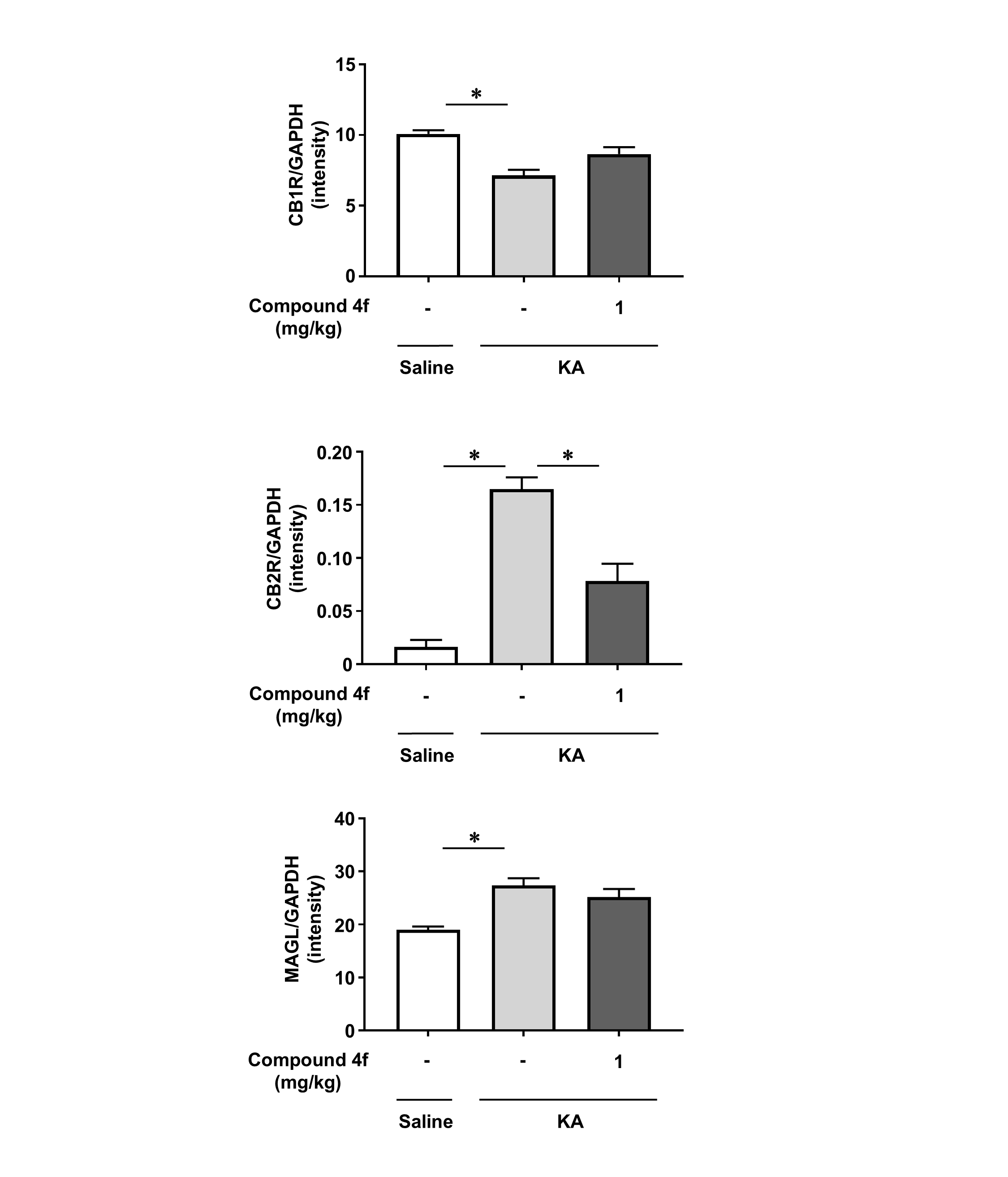

Supplement: S4 Fig — Mean ± SEM (n = 5 for vehicle-treated group of saline-injected mice; n = 8 for vehicle- or compound 4f- treated groups of KA-injected mice). P-values were adjusted for multiple comparisons using the Benjamini–Hochberg false discovery rate. *Adjusted P value ≤ 0.05, compared to saline-injected mice or KA-injected mice. (TIF) [file pone.0312090.s004.tif]
