# Supplementary material for: Compound 4f, a novel brain-penetrant reversible monoacylglycerol inhibitor, ameliorates neuroinflammation, neuronal cell loss, and cognitive impairment in mice with kainic acid-induced neurodegeneration
Source: PLoS One. 2024 Nov 21;19(11):e0312090. doi: 10.1371/journal.pone.0312090 (PMC11581214; doi:10.1371/journal.pone.0312090)
Supplement: S1 Table — In vitro assay profile was performed by Eurofins Cerep PanLabs Taiwan, Ltd. (Taipei, Taiwan). The basic methods employed in this study were adapted from scientific literature to maximize the reliability and reproducibility. Reference standards were used as an integral part of each assay to ensure the validity of the results. Items meeting the criteria for significance (≥50% stimulation or inhibition) are highlighted. bov = bovine; gp = guinea pig; ham = hamster; hum = human; pig = porcine. %Inh.: % of inhibition. %Cont.: % of control. (PDF) [file pone.0312090.s005.PDF]

S1 Table. *In vitro* Assay profile of Compound 4f in Eurofins Cerep Panlabs

- Enzyme and binding assay

Assay name	Species	Conc.	%Inh.
5-Lipoxygenase	hum	10 μM	9
ATPase, Ca^{2+}, Skeletal Muscle, Pig	pig	10 μM	1
ATPase, Na^{+}/K^{+}, Heart, Pig	pig	10 μM	0
Carbonic Anhydrase II	hum	10 μM	-1
Catechol-O-Methyl Transferase (COMT)	pig	10 μM	1
Cholinesterase, Acetyl, ACES	hum	10 μM	-19
Cyclooxygenase COX-1	hum	10 μM	11
Cyclooxygenase COX-2	hum	10 μM	-10
HMG-CoA Reductase	hum	10 μM	-5
Monoamine Oxidase MAO-B	hum	10 μM	50
Nitric Oxide Synthase, Neuronal (nNOS)	rat	10 μM	0
Nitric Oxide Synthetase, Inducible (iNOS)	mouse	10 μM	5
Peptidase, Factor Xa	hum	10 μM	-12
Peptidase, Matrix Metalloproteinase-1 (MMP-1)	hum	10 μM	-5
Peptidase, Matrix Metalloproteinase-7 (MMP-7)	hum	10 μM	4
Peptidase, Matrix Metalloproteinase-13 (MMP-13)	hum	10 μM	3
Peptidase, Metalloproteinase, Neutral Endopeptidase	hum	10 μM	0
Phosphodiesterase PDE10A2	hum	10 μM	-13
Phosphodiesterase PDE3	hum	10 μM	-3
Phosphodiesterase PDE4	hum	10 μM	-5
Phosphodiesterase PDE4D2	hum	10 μM	-1
Phosphodiesterase PDE5	hum	10 μM	-10
Phosphodiesterase PDE6	bov	10 μM	-9
Protein Serine/Threonine Kinase, PKC, Non-Selective	rat	10 μM	-16
Protein Serine/Threonine Kinase, PRKACA (PKA)	hum	10 μM	-23
Protein Serine/Threonine Kinase, ROCK1	hum	10 μM	3
Protein Tyrosine Kinase, EGF Receptor	hum	10 μM	1
Steroid 5α-Reductase	rat	10 μM	-2
Xanthine Oxidase	bov	10 μM	6
Adenosine A_1	hum	10 μM	1
Adenosine A_{2A}	hum	10 μM	-8

Items meeting criteria for significance (≥50% stimulation or inhibition) are highlighted.

Bov = bovine; gp = guinea pig; ham = hamster; hum = human; pig = porcine

%Inh.: % of inhibition

- Enzyme and binding assay (continued)

Assay name	Species	Conc.	%Inh.
Adenosine A_{2B}	hum	10 µM	16
Adrenergic α_1, Non-Selective	rat	10 µM	-2
Adrenergic α_2, Non-Selective	rat	10 µM	19
Adrenergic β_1	hum	10 µM	-1
Adrenergic β_2	hum	10 µM	2
Adrenergic β_3	hum	10 µM	4
Androgen (Testosterone)	hum	10 µM	5
Angiotensin AT_1	hum	10 µM	10
Angiotensin AT_2	hum	10 µM	-4
Bradykinin B_1	hum	10 µM	5
Bradykinin B_2	hum	10 µM	9
Calcium Channel L-Type, Benzothiazepine	rat	10 µM	-10
Calcium Channel L-Type, Dihydropyridine	rat	10 µM	0
Calcium Channel L-Type, Phenylalkylamine	rat	10 µM	-10
Calcium Channel N-Type	rat	10 µM	4
Cannabinoid CB_1	hum	10 µM	19
Cannabinoid CB_2	hum	10 µM	5
Cholecystokinin CCK_1 (CCK_A)	hum	10 µM	0
Cholecystokinin CCK_2 (CCK_B)	hum	10 µM	11
Dopamine D_1	hum	10 µM	-5
Dopamine D_{2L}	hum	10 µM	8
Dopamine D_{2S}	hum	10 µM	3
Dopamine D_3	hum	10 µM	9
Dopamine $D_{4.2}$	hum	10 µM	3
Endothelin ET_A	hum	10 µM	1
Estrogen Receptor (non-selective)	hum	10 µM	-11
$GABA_A$, Chloride Channel, TBOB	rat	10 µM	-6
$GABA_A$, Flunitrazepam, Central	rat	10 µM	-11
$GABA_A$, Muscimol, Central	rat	10 µM	5
$GABA_B$, Non-Selective	rat	10 µM	0
$GABA_{B1A}$	hum	10 µM	4
$GABA_{B1B}$	hum	10 µM	13
Glucocorticoid	hum	10 µM	7
Glutamate, AMPA	rat	10 µM	-2
Glutamate, Kainate	rat	10 µM	2
Glutamate, NMDA, Agonism	rat	10 µM	7

Items meeting criteria for significance (≥50% stimulation or inhibition) are highlighted.

bov = bovine; gp = guinea pig; ham = hamster; hum = human; pig = porcine

%Inh.: % of inhibition

- Enzyme and binding assay (continued)

Assay name	Species	Conc.	%Inh.
Glutamate, NMDA, Glycine	rat	10 µM	1
Glutamate, NMDA, Phencyclidine	rat	10 µM	9
Glycine, Strychnine-Sensitive	rat	10 µM	6
Growth Hormone Secretagogue (GHS, Ghrelin)	hum	10 µM	6
Histamine H_1	hum	10 µM	-6
Histamine H_2	hum	10 µM	-14
Imidazoline I_2, Central	rat	10 µM	24
Insulin	rat	10 µM	3
IP (PGI2)	hum	10 µM	8
Melatonin MT_1	hum	10 µM	-4
Muscarinic M_1	hum	10 µM	8
Muscarinic M_2	hum	10 µM	0
Muscarinic M_3	hum	10 µM	4
Nicotinic Acetylcholine	hum	10 µM	-5
Opiate δ_1 (OP1, DOP)	hum	10 µM	8
Opiate κ(OP2, KOP)	hum	10 µM	6
Opiate µ(OP3, MOP)	hum	10 µM	2
Potassium Channel [K_{ATP}]	ham	10 µM	11
Potassium Channel [SK_{CA}]	rat	10 µM	-6
Progesterone PR-B	hum	10 µM	8
Serotonin (5-Hydroxytryptamine) $5\text{-}HT_{1A}$	hum	10 µM	5
Serotonin (5-Hydroxytryptamine) $5\text{-}HT_{2A}$	hum	10 µM	2
Serotonin (5-Hydroxytryptamine) $5\text{-}HT_{2B}$	hum	10 µM	81
Serotonin (5-Hydroxytryptamine) $5\text{-}HT_{2C}$	hum	10 µM	10
Serotonin (5-Hydroxytryptamine) $5\text{-}HT_3$	hum	10 µM	4
Serotonin (5-Hydroxytryptamine) $5\text{-}HT_4$	gp	10 µM	-2
Sigma, Non-Selective	gp	10 µM	62
Sodium Channel, Site 2	rat	10 µM	10
Tachykinin NK_1	hum	10 µM	3
Tachykinin NK_2	hum	10 µM	0
Tachykinin NK_3	hum	10 µM	4
Transporter, Dopamine (DAT)	hum	10 µM	30
Transporter, GABA	rat	10 µM	-12
Transporter, Norepinephrine (NET)	hum	10 µM	20
Transporter, Serotonin (5-Hydroxytryptamine) (SERT)	hum	10 µM	9

Items meeting criteria for significance (≥50% stimulation or inhibition) are highlighted.

bov = bovine; gp = guinea pig; ham = hamster; hum = human; pig = porcine

%Inh.: % of inhibition

- Enzyme and binding assay (continued)

Assay name	Species	Conc.	%Inh.
Transporter, Vesicular Monoamine (Non-Selective)	hum	10 μM	-8
Vasopressin V_{1A}	hum	10 μM	-1
Vasopressin V_2	hum	10 μM	6

Items meeting criteria for significance (≥50% stimulation or inhibition) are highlighted.

bov = bovine; gp = guinea pig; ham = hamster; hum = human; pig = porcine

%Inh.: % of inhibition

- Cellular functional assay

Assay name	Species	Conc.	%Cont.
**Neurotensin-1 (NT1) (agonistic effect)**	**hum**	**10 μM**	**6.5**
**5-HT7 (agonistic effect)**	**hum**	**10 μM**	**-1.5**
**TRPV1 (agonistic effect)**	**hum**	**10 μM**	**14.6**
**TRMP8 (antagonistic effect)**	**hum**	**10 μM**	**-27.3**

Results are expressed as a percent of control agonist response or inverse agonist response.

%Cont.: % of control
