# Supplementary material for: Compound 4f, a novel brain-penetrant reversible monoacylglycerol inhibitor, ameliorates neuroinflammation, neuronal cell loss, and cognitive impairment in mice with kainic acid-induced neurodegeneration
Source: PLoS One. 2024 Nov 21;19(11):e0312090. doi: 10.1371/journal.pone.0312090 (PMC11581214; doi:10.1371/journal.pone.0312090)
Supplement: S2 Table — P value for KA-injected mice treated with compound 4f compared to KA-injected mice treated with the vehicle. Ratio (KA + vehicle/KA + compound 4f): Number of genes significantly changed by compound 4f among those genes changed by KA (FC ≤ -1.2 or ≥ 1.2)/number of genes changed by KA (FC ≤ -1.2 or ≥ 1.2). (PDF) [file pone.0312090.s006.PDF]

S2 Table. Compound 4f Restores the Expression of Several Genes Changed by Kainic Acid (KA) in Neuroinflammation, γ-Aminobutyric Acid (GABA) Receptor, Glutamate Receptor, and Endocannabinoid Neuronal Synapse Pathways.

Pathway	- log (p-value)	Ratio (KA+veihcle/KA+compound 4f)	Genes
Neuroinflammation signaling	20.500	60/99	**Cxcl10**, **Ccl5**, **Irf7**, **Cybb**, **Ccl12**, **H2-Aa**, **H2-Q7**, **H2-Q6**, **H2-Ab1**, **H2-Eb1**, **Tlr2**, **Birc5**, **Stat1**, **Naip2**, **B2m**, **H2-T23**, **Tlr1**, **Tlr13**, **Pycard**, **Tyrobp**, **Icam1**, **Trem2**, **Ncf1**, **Nlrp3**, **Tlr7**, **H2-DMb1**, **Tnfrsf1a**, **Pla2g4a**, **H2-Ob**, **Naip5**, **Casp8**, **Pik3r5**, **Ncf2**, **Irak4**, **Tgfbr2**, **Tgfb1**, **Hmox1**, **Myd88**, **Birc3**, **Plcg2**, **Cd86**, **Tlr9**, **Ikbke**, **Pik3cg**, **Vcam1**, **Tlr4**, **Tlr3**, **Cx3cr1**, **Csf1r**, **Tirap**, **Nfe2l2**, **Pik3r6**, **Nfatc1**, **Gjb1**, **Ripk1**, **Ticam1**, **Cflar**, **Tgfbr1**, **P2rx7**, **Il1r1**, H2-Oa, Nfkb2, Ticam2, Jak3, Cd40, Syk, Fos, Tlr6, Tlr12, Pla2g5, Casp1, Nos2, Fas, Acvr1c, Ifngr1, Mapk12, Il12a, Bace2, Irak3, Ager, Il6ra, Casp3, Nfkb1, Rela, Nfatc4, Bcl2, Akt2, Bdnf, Jun, Mapk6, Acvr1, S100b, Nfatc3, Pik3cd, Jak2, Gabrb1, Slc1a3, Irf3, Ide
GABA receptor signaling	6.730	9/39	**Gabra3**, **Gad2**, **Gad1**, **Kcnh2**, **Dnm1**, **Slc32a1**, **Cacng3**, **Gabra1**, **Slc6a1**, Cacna1a, Ubb, Gabrg2, Cacna2d3, Gabrg1, Cacnb3, Adcy2, Gabrg3, Cacnb1, Cacng2, Cacna1i, Gabbr2, Cacnb4, Gabrb3, Cacnb2, Nsf, Cacna2d1, Cacng7, Cacng8, Cacna1c, Slc6a11, Cacna1b, Cacna1e, Gabrb2, Kcnq3, Abat, Adcy9, Gabra5, Adcy1, Gabrd
Glutamate receptor signaling	3.810	5/27	**Homer2**, **Grik3**, **Gria3**, **Homer1**, **Gria4**, Slc17a7, Grid1, Gng7, Grik4, Slc1a1, Gng2, Gls, Grin2b, Grip1, Grin2d, Dlg4, Grin3a, Homer3, Calm3, Grm1, Gria1, Gria2, Grin1, Camk4, Grm5, Grin2a, Slc17a8
Endocannabinoid neuronal synapse	< 1.30103 (*n.s.*)	5/48	**Itpr1**, **Gria3**, **Cacng3**, **Rims1**, **Gria4**, Cacna1a, Cacna2d3, Cacnb3, Adcy2, Cacnb1, Cacng2, Plcb1, Cacna1i, Mapk8, Gng7, Cacnb4, Gnaq, Cacnb2, Gng2, Cacna2d1, Cacng7, Cacng8, Kcnj3, Cacna1c, Kcnj6, Cacna1b, Grin2b, Mapk10, Ppp3cb, Faah, Grin2d, Cacna1e, Grin3a, Gnao1, Plch2, Dagla, Grm1, Gria1, Gria2, Grin1, Grm5, Cnr1, Gnai1, Ppp3ca, Adcy9, Ppp3r1, Grin2a, Adcy1

p-value for KA-injected mice treated with compound **4f** compared to KA-injected mice treated with vehicle

Ratio (KA + veihcle/KA + compound **4f**): Number of genes significantly changed by compound 4f among those changed by KA (FC ≤ -1.2 or ≥ 1.2)/number of genes changed by KA (FC ≤ -1.2 or ≥ 1.2)

Bold: genes significantly changed by compound **4f**

n.s.: not significant
