# Supplementary material for: Compound 4f, a novel brain-penetrant reversible monoacylglycerol inhibitor, ameliorates neuroinflammation, neuronal cell loss, and cognitive impairment in mice with kainic acid-induced neurodegeneration
Source: PLoS One. 2024 Nov 21;19(11):e0312090. doi: 10.1371/journal.pone.0312090 (PMC11581214; doi:10.1371/journal.pone.0312090)

Schematic diagram illustrating experimental design of KA-induced neurodegeneration model

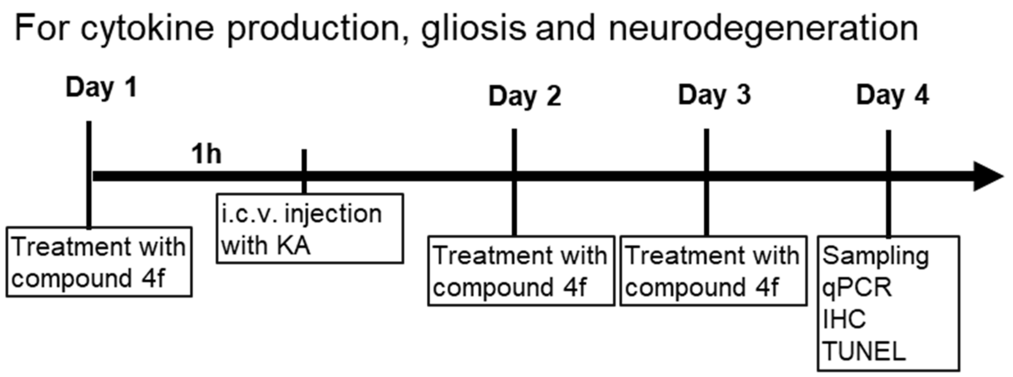

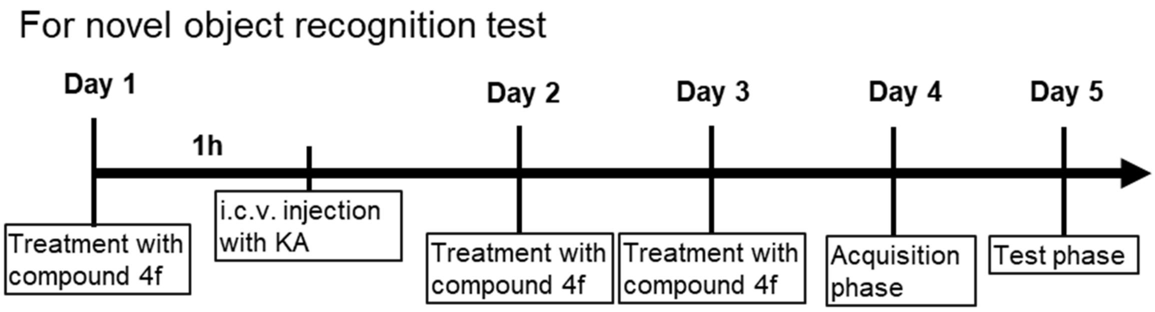

Supplement: S1 File — (PDF) [file pone.0312090.s007.pdf]
