# Supplementary material for: Compound 4f, a novel brain-penetrant reversible monoacylglycerol inhibitor, ameliorates neuroinflammation, neuronal cell loss, and cognitive impairment in mice with kainic acid-induced neurodegeneration
Source: PLoS One. 2024 Nov 21;19(11):e0312090. doi: 10.1371/journal.pone.0312090 (PMC11581214; doi:10.1371/journal.pone.0312090)
Supplement: S2 File — (PDF) [file pone.0312090.s008.pdf]

Experimental procedures in supporting information

In vitro assay profile

In vitro assay profile was performed by Eurofins Cerep PanLabs Taiwan, Ltd (Taipei, Taiwan). Basic methods employed in this study have been adapted from the scientific literature to maximize reliability and reproducibility. Reference standards were run as an integral part of each assay to ensure the validity of the results obtained.

*Effects of compound **4f** on seizure score in KA-injected mice*

Compound **4f** (0.1, 0.3, or 1 mg/kg) was administered to seven-week-old mice. One hour after compound **4f** administration, mice were anesthetized with pentobarbital sodium (50 mg/kg, intraperitoneal, Somnopentyl®, Kyoritsu Seiyaku, Tokyo, Japan) and fixed to a stereotaxic apparatus (Kopf Instruments, Tujunga, CA, USA). For intracerebroventricular (i.c.v.) administration of KA (0.2 μg), an injection cannula was implanted into the left lateral ventricle (0.2 mm posterior to bregma, 1.0 mm lateral to the midline, 2.0 mm depth from the skull surface). Two hours after the KA injection, each mouse was observed for 5 min by a blinded observer for measuring the behavioral seizure score using a modified Racine scale (Cipriani et al., 2015): grade (0) normal; (1) immobilization; (2) forelimb and/or tail extension, rigid posture; (3) repetitive movements, head bobbing, and

gnawing; (4) rearing and falling; (5) continuous rearing and falling; (6) severe tonic-clonic seizure with loss of postural control; and (7) death in the first 2 h.

Reference for Supporting Information

Cipriani, R., Chara, J.C., Rodríguez-Antigüedad, A., Matute, C., 2015. FTY720 attenuates excitotoxicity and neuroinflammation. J. Neuroinflammation. 12:86.
